# IER5 generates a novel hypo-phosphorylated active form of HSF1 and contributes to tumorigenesis

**DOI:** 10.1038/srep19174

**Published:** 2016-01-12

**Authors:** Yoshinori Asano, Tatsuya Kawase, Atsushi Okabe, Shuichi Tsutsumi, Hitoshi Ichikawa, Satoko Tatebe, Issay Kitabayashi, Fumio Tashiro, Hideo Namiki, Tadashi Kondo, Kentaro Semba, Hiroyuki Aburatani, Yoichi Taya, Hitoshi Nakagama, Rieko Ohki

**Affiliations:** 1 Division of Rare Cancer Research, National Cancer Center Research Institute, Tsukiji 5-1-1, Chuo-ku, Tokyo 104-0045, Japan; 2 Graduate School of Advanced Science and Engineering, Waseda University, 3-4-1 Okubo, Shinjuku-ku, Tokyo, 169-8555, Japan; 3 Radiobiology Division, National Cancer Center Research Institute, Tsukiji 5-1-1, Chuo-ku, Tokyo 104-0045, Japan; 4 Department of Biological Science and Technology, Faculty of Industrial Science and Technology, Tokyo University of Science, Niijuku 6-3-1, Katsushika-ku, Tokyo 125-8585, Japan; 5 Genome Science Division, Research Center for Advanced Science and Technology, The University of Tokyo, 4-6-1 Komaba, Meguro-ku, Tokyo 153-8904, Japan; 6 Department of Clinical Genomics, National Cancer Center Research Institute, Tsukiji 5-1-1, Chuo-ku, Tokyo 104-0045, Japan; 7 Division of Hematological Malignancy, National Cancer Center Research Institute, Tsukiji 5-1-1, Chuo-ku, Tokyo 104-0045, Japan; 8 Division of Cancer Development System, National Cancer Center Research Institute, Tsukiji 5-1-1, Chuo-ku, Tokyo 104-0045, Japan

## Abstract

The transcription factors HSF1 and p53 both modulate the stress response, thereby protecting and facilitating the recovery of stressed cells, but both have the potential to promote tumor development. Here we show that a p53 target gene, *IER5*, encodes an activator of HSF1. IER5 forms a ternary complex with HSF1 and the phosphatase PP2A, and promotes the dephosphorylation of HSF1 at numbers of serine and threonine residues, generating a novel, hypo-phosphorylated active form of HSF1. *IER5* is also transcriptionally upregulated in various cancers, although this upregulation is not always p53-dependent. The *IER5* locus is associated with a so-called super enhancer, frequently associated with hyperactivated oncogenes in cancer cell lines. Enhanced expression of IER5 induces abnormal HSF1 activation in cancer cells and contributes to the proliferation of these cells under stressed conditions. These results reveal the existence of a novel IER5-mediated cancer regulation pathway that is responsible for the activation of HSF1 observed in various cancers.

The *p53* tumor suppressor gene is one of the most frequently mutated genes in human cancer, and the loss of functional p53 is a prerequisite for oncogenesis in many cancers^1^. p53 functions as a transcriptional activator that induces various genes involved in the suppression of tumorigenesis^2^,^3^,^4^. These target genes modulate growth arrest, DNA repair, cell death, metabolism, cellular homeostasis and a variety of other functions. Under conditions of severe stress, p53 induces apoptosis and/or senescence to eliminate cells that are irreparably damaged. However, under conditions of mild stress, p53 instead elicits a survival response, induces genes that are involved in cell-cycle arrest, DNA repair and regulation of metabolism, and thereby acts to protect cells and facilitate their recovery from stress^5^. This p53-mediated survival response may suppress tumorigenesis in normal cells, but may have the potential to promote tumor development in cells that otherwise would not recover or be repaired. For example, among the p53 target genes are several that adapt cells to metabolic changes such as nutrient deprivation and ROS. This function could enable cancer cells to survive under harsh conditions and thereby contribute to cancer progression^4^.

HSF1 is a constitutively expressed transcriptional activator, a master regulator of the heat shock response, and is post-translationally activated following heat shock^6^. Under non-stressed conditions, HSF1 exists as a monomer in complex with HSP90, which negatively regulates its activity. Heat shock induces the dissociation of HSF1 from HSP90, allowing HSF1 to multimerize into a trimer, which can accumulate in the nucleus and bind DNA. Activated HSF1 induces a number of HSF1 target genes including the *HSP* family of genes, which allow the cell to adapt and recover from stress. Moreover, it has been recently reported that HSF1 can promote tumorigenesis. HSF1 is constitutively activated in cancer tissues and higher HSF1 activity is related to poorer prognosis of cancer patients^7^. HSF1 has also been shown to transactivate a variety of genes involved in tumor progression^8^. A study of HSF1-deficient mice showed that tumorigenesis driven by oncogenic ras or mutant p53 is HSF1-dependent^9^. The ability of HSF1 to promote tumor development is critically dependent on its ability to upregulate HSF1 target genes, including the *HSP* family of genes, which facilitate the survival of cancer cells and their adaptation to hostile conditions^8^. Thus, HSF1 resembles p53 in its ability to both protect stressed cells and in its potential to promote tumor formation.

Previously, we searched for novel genes regulating tumorigenesis by analyzing p53 target genes^10^. We introduced a temperature-sensitive p53 mutant into the p53-null Saos2 cell line, and looked for genes that were induced upon temperature shift to the permissive temperature. In addition, to identify genes modulated directly by p53, we performed Chip-seq analysis using HCT116 cells, which contain wild-type p53. From these analyses, we identified several putative p53 target genes, i.e., genes that are both induced by p53 and to which p53 binds^11^,^12^. Here, we report that one of these p53 target genes, *IER5*, is a hitherto unknown activator of HSF1 that regulates HSF1 activity via a novel mechanism. We show that IER5 facilitates the dephosphorylation of several serine and threonine residues in HSF1, generating a hypo-phosphorylated active form of HSF1 that is distinct from that induced by heat shock. The *IER5* gene has been known as an immediate-early gene induced by various growth-promoting stimuli, and is overexpressed in various cancers^13^,^14^. We observe that depletion of IER5 in cancer cells results in decreased HSF1 activity. Furthermore, IER5-mediated activation of HSF1 is required for anchorage-independent cell growth of cancer cells. These results collectively indicate that IER5 has oncogenic potential and is responsible for the activation of HSF1 in cancer.

## Results

### The *IER5* gene is a p53 target gene

**Figure 1:**
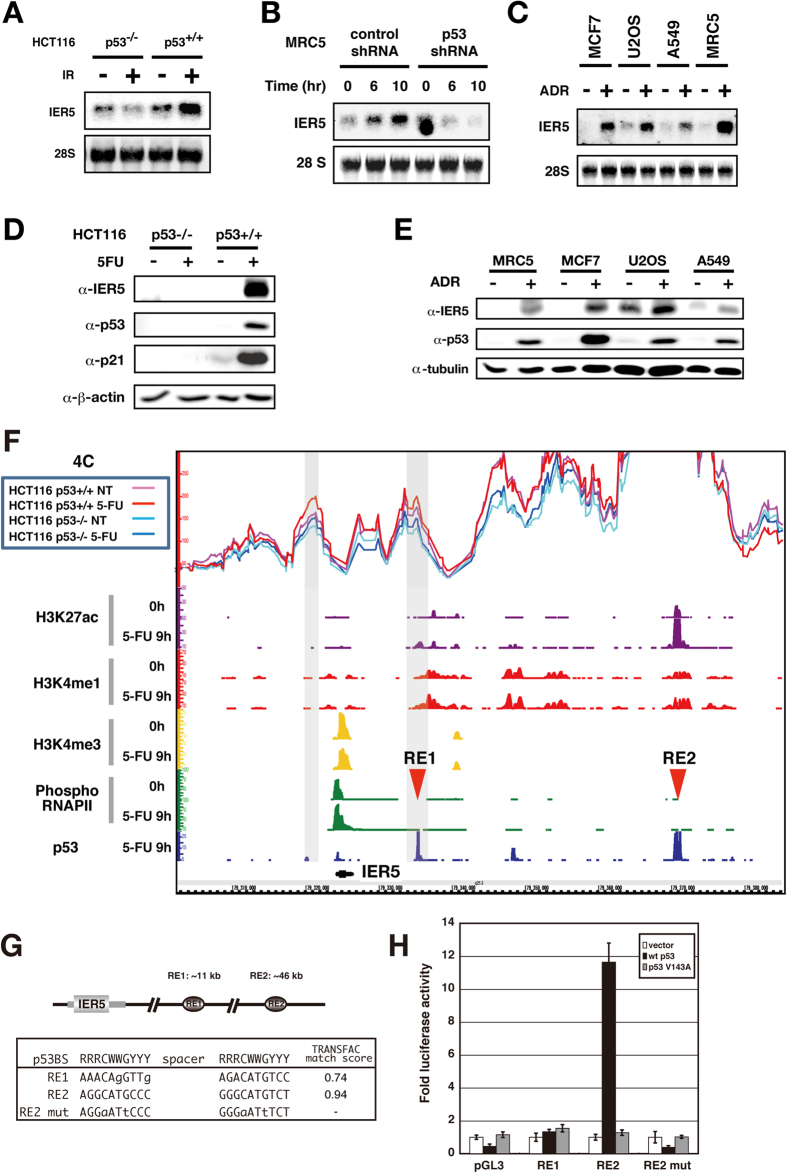
The *IER5* gene is a p53 target gene. (**A–C**) *IER5* expression was analyzed by Northern blotting. HCT116 *p53* (+/+), HCT116 *p53* (−/−) were subjected to γ-ray irradiation (20 Gy) (**A**). Normal human fibroblasts (MRC5 cells) transfected with control lentivirus or lentivirus expressing p53 shRNA were subjected to γ-ray irradiation (30 Gy) (**B**). Cell lines carrying wild-type p53 were treated with Adriamycin (1 μM) for 24 hrs (MCF7, U2OS and MRC5) or 19 hrs (A549) (**C**). (**D**) HCT116 *p53* (+/+), HCT116 *p53* (−/−) were treated with 5-FU (0.038 mM) for 24 hrs. Expression of IER5, p53 and p21 (representative p53 target gene) were analyzed by Western blotting. (**E**) Cell lines carrying wild-type p53 were treated with Adriamycin (1 μM) for 24 hrs (MRC5, MCF7, U2OS) or 19 hrs (A549). Expression of IER5 and p53 were analyzed by Western blotting. (**F**) Genomic locus of IER5 is shown together with the ChIP-seq and 4C-seq results. HCT116 *p53* (+/+) cells treated with or without 5-FU were used for ChIP-seq analysis. ChIP-seq analyses were performed using antibodies against p53, H3K27ac, H3K4me1, H3K4me3 and phospho-RNAP II. Two p53 binding sites RE1 (11 kb downstream) and RE2 (46 kb downstream) were identified. Chromatin interaction was detected by 4C-seq analysis in HCT116 *p53*+/+ and HCT116 *p53*−/− cells with or without 5-FU. Primers were designed around RE2, and the genomic regions interacting with RE2 were determined. (**G**) The positions and nucleotide sequences of RE1 and RE2. (**H**) Double-stranded synthetic oligonucleotides containing RE1, RE2 or mutant RE2 (the sequences are shown in **G**) were inserted into the luciferase reporter plasmid containing a minimal promoter. The assay was performed 24 hrs post-transfection. The experiment was run in triplicate, and data are represented as the mean-fold activation ±SD.

In order to discover potentially novel, cancer associated genes, we previously undertook a comprehensive effort to identify p53 target genes, and subsequently analyzed several whose functions were unknown^10^,^11^,^12^,^15^,^16^. This study focuses on one of these genes, *IER5*. *IER5* mRNA (Fig. 1A–C) and protein (Fig. 1D,E) are induced by DNA-damaging reagents such as 5-FU, γ-ray and Adriamycin (doxorubicin). In addition, induction of *IER5* mRNA and protein by these treatments is p53-dependent (Fig. 1A,B,D).

The *IER5* gene promoter region was analyzed by ChIP-seq analysis of 5-FU treated and untreated HCT116 *p53*^+/+^ cells. We detected H3K4 tri-methylation surrounded by H3K4 mono-methylation at the *IER5* gene promoter region, indicating that *IER5* is actively transcribed in both unstressed and stressed conditions (Fig. 1F). Furthermore, we detected phospho-RNA polymerase II binding to the *IER5* promoter and this binding increased upon 5-FU treatment, consistent with enhanced transcription of *IER5*. ChIP-seq analysis identified two p53 binding sites 11 kb (RE1) and 46 kb (RE2) downstream of the *IER5* gene (Fig. 1F). p53 binding to RE2 was strong, while binding to RE1 was relatively weak. In addition, H3K27 acetylation was detected at RE2, and was strongly increased by 5-FU treatment, showing that RE2 is an active enhancer that is highly activated upon DNA damage. In contrast, we could not detect H3K27 acetylation at RE1, suggesting that it is not an active enhancer, even though it does bind p53. We found sequences highly similar to the p53 consensus binding sequence at the center of RE2 (TRANSFAC match score; 0.94) but not in RE1 (TRANSFAC match score; 0.74, Fig. 1G). We generated DNA oligonucleotides representing RE1 and RE2, and created a version of RE2 mutated in the p53 consensus sequence (RE2 mut; sequences shown in Fig. 1G), and cloned each upstream of a luciferase reporter gene containing a minimal promoter. As shown in Fig. 1H, p53 strongly activated a reporter containing RE2, but not RE2 mut or RE1. The lack of RE1 enhancer activity is consistent with the weak binding of p53 and the lack of H3K27 acetylation at RE1 seen with ChIP-seq, indicating that RE1 is not an active enhancer.

We further performed 4C-seq analysis to identify chromatin regions that might interact with RE2. As shown in Fig. 1F, we detected chromatin interaction between RE2 and RE1, and between RE2 and the *IER5* gene promoter region, in both HCT116 *p53* +/+ and −/− cells. The interactions between the RE2 and the *IER5* promoter region seem to be stabilized by 5-FU treatment in HCT116 *p53* +/+ cells but not in HCT116 *p53* −/− cells. In contrast, the interactions between RE2 and RE1 seem to be stronger in HCT116 *p53* +/+ cells compared to HCT116 *p53* −/− cells, but are unchanged by 5-FU treatment. The RE2 region also interacts with several regions that are positive for H3K4me1. Collectively, these results show that RE2 is localized in the vicinity of the *IER5* promoter when a higher order chromatin structure is formed, and identifies RE2 as the p53-responsive element of the *IER5* gene. In addition, since RE1 and RE2 were constitutively associated in the absence of 5-FU treatment in HCT *p53* +/+ cells, p53 binding to both RE1 and RE2 may facilitate the formation of higher-order chromatin structure at this locus.

### *IER5* transcription is driven by super-enhancers in cancer cells, leading to enhanced IER5 expression

**Figure 2:**
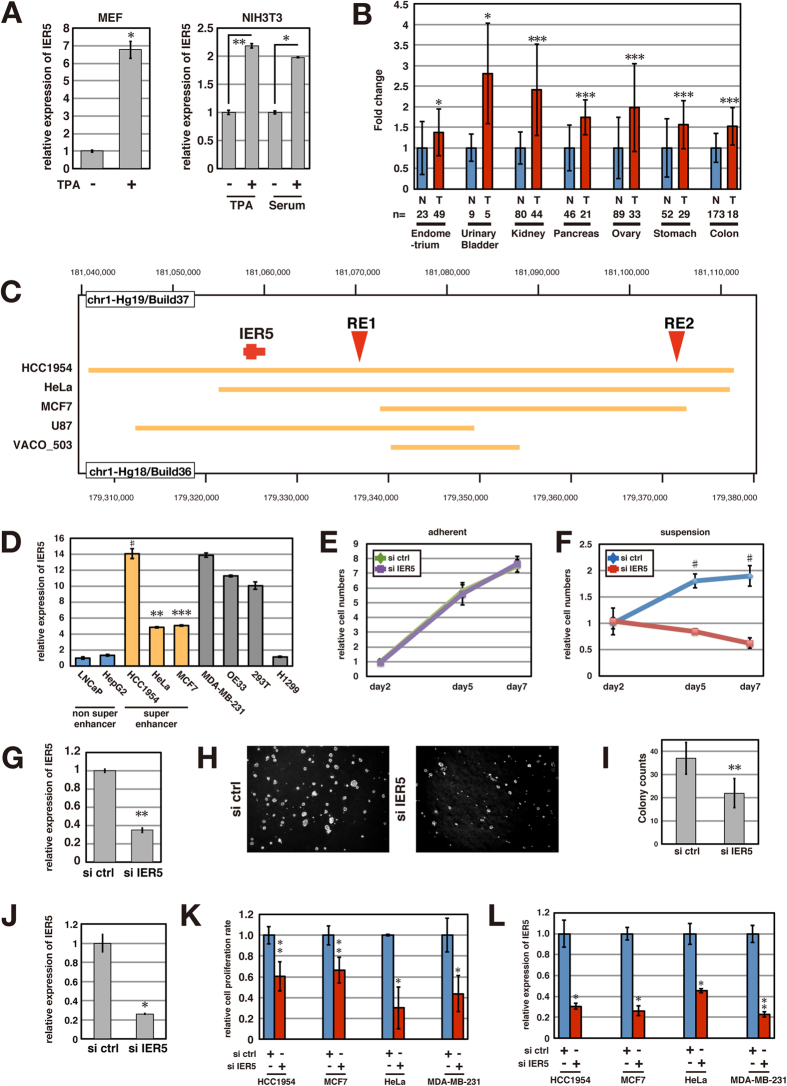
*IER5* mRNA expression is increased in various cancers and IER5 is required for anchorage-independent cancer cell proliferation. (**A**) Mouse embryonic fibroblasts and NIH3T3 cells were treated with TPA (160 ng/ml) for 30 min. NIH3T3 cells were also cultured with 0.5% FBS DMEM medium overnight and then stimulated by 10% FBS DMEM for 2 hrs. Expression of *IER5* mRNA was analyzed by quantitative RT-PCR. (*p < 0.05, **p < 0.01). (**B**) *IER5* mRNA expression levels were analyzed in the indicated cancers and their corresponding normal tissues using the Gene Logic SCIANTIS database. (*p < 0.05, ***p < 0.001). (**C**) The *IER5* gene is associated with super-enhancers in various cancer cell lines. The locus of the *IER5* gene is shown with both of the human genome reference versions, Hg19/Build37 (upper) and Hg18/Build36 (lower). The regions identified as containing super enhancers by Hnisz *et al.* are shown by yellow lines^17^. (**D**) *IER5* mRNA expression in various cancer cell lines. Significance of difference was calculated between non-super enhancer cell lines and super enhancer cell lines. (**p < 0.01, ***p < 0.001, ^#^p < 0.0001). (**E–G**) OE33 cells were plated in adherent (**E**) or suspension (**F**) 96-well culture plates, and control or IER5-targeting siRNAs were introduced. Cell growth assays were performed at the indicated days. Mean relative cell numbers ±SD are shown. *IER5* mRNA expression was analyzed by quantitative RT-PCR 48 hrs post-transfection (**G**). (**p < 0.01, ^#^p < 0.0001). (**H–J**) Anchorage-independent growth in soft agar was analyzed. Control or IER5-targeting siRNAs were introduced into OE33 cells. The representative images (**H**) and the mean numbers of colonies ±SD are shown (**I**). Expression of *IER5* (**J**) was analyzed by quantitative RT-PCR 48 hrs post-transfection. (*p < 0.05, **p < 0.01). (**K,L**) Cell growth assays were performed using the indicated cell lines. Cells were plated in suspension culture plates and analyzed as in (**F**). Cell growth was analyzed 7 days (HCC1954, MCF7 and HeLa cells) or 8 days (MDA-MB-231 cells) post-transfection (**K**). *IER5* mRNA expression was analyzed by quantitative RT-PCR 48 hrs post-transfection (**L**). (*p < 0.05, **p < 0.01).

Originally, *IER5* was reported as a gene induced by growth-promoting stimuli and that is highly expressed in cervical cancers^13^,^14^. Consistent with this, we observed that IER5 could be induced by various mitogenic stimuli such as PMA, serum and ionomycin (Fig. 2A and S1A). In addition, *IER5* upregulation and overexpression has been observed in a number of cancers (Fig. 2B and S1B). These results collectively suggest that various growth-promoting and oncogenic signals can induce expression of IER5.

Recently, it was reported that many cancer cells acquire so-called “super-enhancers” at key driver oncogenes, and other genes important for tumor pathogenesis^17^,^18^. Since enhanced *IER5* expression is observed in many cancers, we asked whether the *IER5* gene locus might also be associated with a super-enhancer in cancer cells. A comprehensive analysis of super-enhancers in 6 blood cancer-derived cell lines and 12 solid cancer-derived cell lines has been recently reported^17^. The *IER5* gene was included in this analysis, and as shown in Fig. S1C, there was no association of the *IER5* gene with a super enhancer in the 6 blood cancer-derived cell lines we examined, but there was an association in 5 of the 12 solid cancer-derived cell lines, regardless of p53 status. These super enhancers were localized around the *IER5* gene locus including the RE1 and RE2 regions (Fig. 2C). This remarkably high incidence of super-enhancer association with the *IER5* gene in cancer cell lines supports the idea that *IER5* may be an important gene in tumor pathogenesis.

### IER5 is required for anchorage-independent cancer cell growth

In order to analyze the function of IER5 in tumorigenesis, we examined the effect of IER5 expression on the growth of cancer cells. First, we analyzed the expression levels of *IER5* in several cancer cell lines including cell lines identified as having or not having *IER5*-associated super enhancers: HCC1954, HeLa, MCF7, LNCaP, and HepG2 (Fig. 2D). Cell lines that have super enhancers at the *IER5* locus had higher *IER5* mRNA expression compared to cell lines without them. We took the OE33 esophageal cancer cell line, as it expressed relatively high levels of *IER5*, and analyzed the effect of IER5 knockdown under both adherent (Fig. 2E) and suspension (Fig. 2F) cell culture conditions. IER5 knockdown was confirmed as shown in Fig. 2G. We found that decreased *IER5* expression had no effect on growth in adherent cultures, but had a significant effect in suspension cultures. In addition, IER5 knockdown significantly decreased colony formation in soft agar (Fig. 2H,I). IER5 knockdown was confirmed as shown in Fig. 2J. We next analyzed the effect of IER5 knockdown in four of the other cancer cell lines, and saw that IER5 had a significant effect on the growth in each line under suspension culture conditions (Fig. 2K). IER5 knockdown was confirmed as shown in Fig. 2L. These data collectively indicate that IER5 has an important function in anchorage-independent proliferation of cancer cells.

### IER5 induces transcription of *HSP* family genes

**Figure 3:**
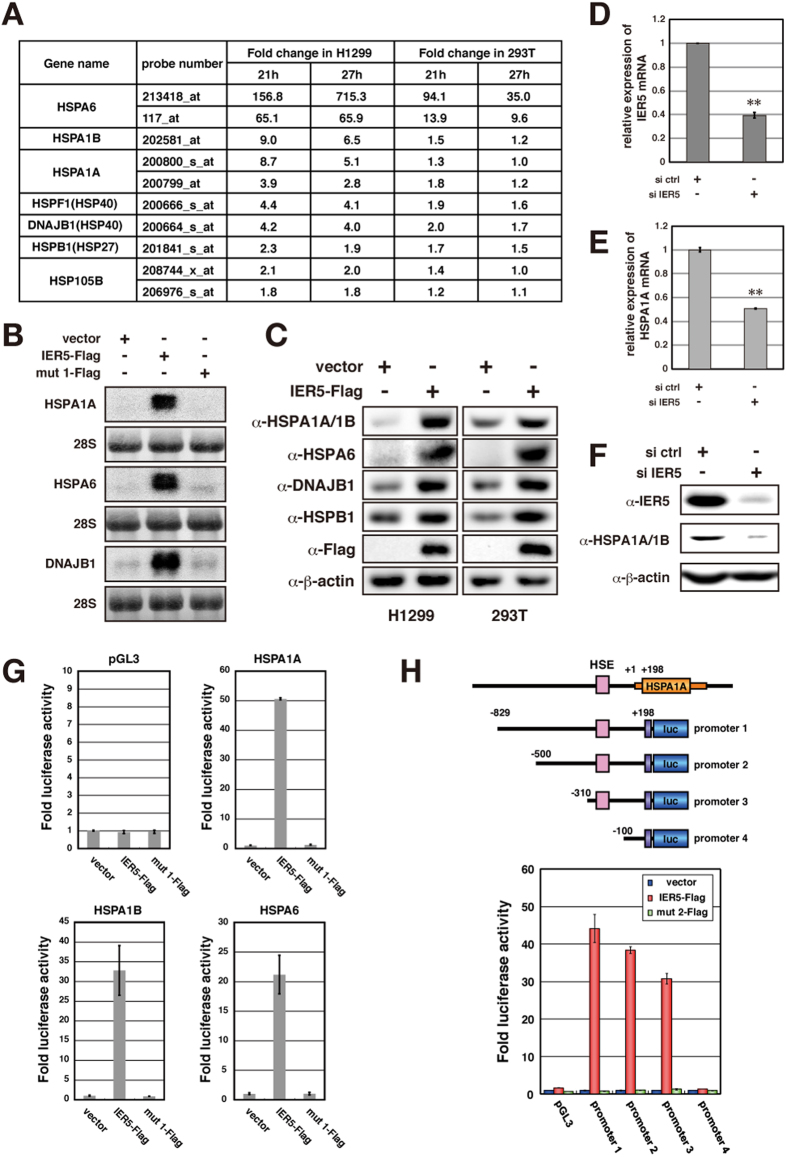
*HSP* family genes are induced by IER5. (**A**) H1299 or 293T cells were transfected with control vector or an IER5 expression vector. Cells were harvested 21 hrs or 27 hrs post-transfection and microarray expression analysis was performed. The table shows the *HSP* family genes, among the genes induced by IER5. (**B**) H1299 cells were transfected with control, IER5-Flag or mutant IER5-Flag expression vectors (representative image of mut 1 is shown in Fig. S1). Cells were harvested 27 hrs post-transfection, and mRNA expressions of the *HSP* family genes were analyzed by Northern blotting. (**C**) H1299 and 293T cells were transfected with control vector or IER5-Flag expression vector, and cells were harvested 24 hrs post-transfection. Expressions of the HSP family proteins were analyzed by Western blotting. (**D–F**) Control or IER5-targeting siRNAs were introduced into OE33 cells. Cells were harvested 52 hrs post-transfection. Expression of IER5 (**D,F**) and HSPA1A (**E,F**) were analyzed by quantitative RT-PCR (**D,E**) and Western blotting (**F**). (**p < 0.01). (**G**) The promoter regions of *HSPA1A*, *HSPA1B* and *HSPA6* were inserted into the luciferase reporter plasmid containing a minimal promoter, and assayed 24 hrs post-transfection. Experiments were run in triplicate, and data are represented as the mean-fold activation ±SD. (**H**) Serially deleted regions of the *HSPA1A* promoter were analyzed as in (**G**). Numbers indicate the position of the 5′ most nucleotide relative to the transcription initiation site. A heat shock element (HSE), to which HSF1 binds, was found between positions −132 and −109.

We next undertook a comprehensive gene expression analysis in cells expressing IER5 using gene microarrays. As shown in Fig. 3A, several genes from the *HSP* family were modestly or highly induced by IER5 expression. These results were further confirmed by Northern blotting (Fig. 3B) and found to require intact N- and C- termini of IER5 (Fig. S2). Upregulation of *HSP* family mRNA expression by IER5 was accompanied by markedly enhanced HSP family protein expression (Fig. 3C). Conversely, siRNA suppression of endogenous IER5 resulted in decreased *HSP* family mRNA and protein expression (Fig. 3D–F).

We next employed a luciferase promoter reporter assay to analyze whether IER5 activates the promoters of *HSP* family genes. As shown in Fig. 3G, the *HSPA1A*, *HSPA1B* and *HSPA6* gene promoters were strongly activated by IER5. In order to identify the IER5-responsive element(s), we generated a series of serially deleted *HSPA1A* promoter regions and cloned these upstream of the luciferase reporter gene. This analysis indicated that the region between −310 and −100 relative to the transcription initiation site of the *HSPA1A* gene mediates IER5 responsiveness (Fig. 3H). Analysis of the sequences within this region revealed the presence of HSF1 transcription factor binding sites, so-called heat shock elements (HSEs).

### IER5 activates HSF1

**Figure 4:**
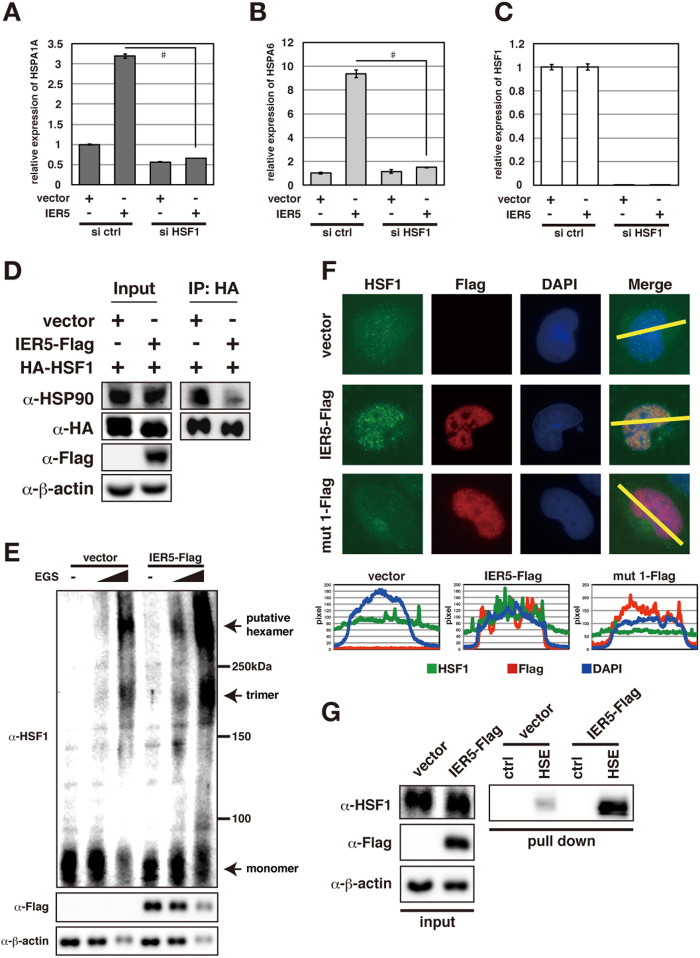
IER5 is a novel activator of HSF1. (**A–C**) Control or HSF1-targeting siRNAs were introduced into H1299 cells. Subsequently, cells were transfected with control vector or an IER5 expression vector 24 hrs post-siRNA transfection. Cells were harvested 24 hrs post-DNA transfection. Expression of *HSPA1A* (**A**), *HSPA6* (**B**) and *HSF1* (**C**) mRNAs were analyzed by quantitative RT-PCR. (^#^p < 0.0001). (**D**) 293T cells were transfected with HA-HSF1 and control vector or IER5-Flag. Cells were harvested 24 hrs post-transfection. Cell lysates were crosslinked by DSP (1 mg/ml), and immunoprecipitated using anti-HA antibody. HSP90 binding to HA-HSF1 was detected by Western blotting. (**E**) H1299 cells were transfected with control vector or IER5-Flag expression vector. Cells were harvested 25 hrs post-transfection. Whole cell lysates were crosslinked with EGS. HSF1 and IER-Flag expression was analyzed by Western blotting. (**F**) H1299 cells were transfected with control vector, IER5-Flag, mut 1-Flag expression vector. Cells were harvested 24 hrs post-transfection. Subcellular localizations of endogenous HSF1 (detected using anti-HSF1 antibody), IER5-Flag and mut 1 (detected using anti-Flag antibody) were analyzed. HSF1 and IER5 expression levels were quantitated and shown at the bottom. (**G**) HSF1 binding to Heat Shock Elements (HSEs) was analyzed by streptavidin pull-down assay. Biotinylated control or HSE-containing DNA probes were bound to streptavidin-coated beads and mixed with control or IER5-expressing cell lysates. Bead-bound HSF1 was denatured in Laemmli buffer and analyzed by Western blotting.

HSF1 binds to HSEs within the promoters of *HSP* family genes and induces their transcription in response to stresses such as heat stress^19^. As we found an HSE within the IER5-responsive region of the *HSPA1A* gene promoter, we analyzed whether induction of HSP family genes by IER5 requires HSF1. As shown in Fig. 4A–C, siRNA suppression of endogenous HSF1 abrogated the induction of these *HSP* family genes by IER5, showing that IER5 upregulation of HSP family genes is dependent on HSF1. HSF1 activation can be regulated at two steps: HSF1 oligomerization and nuclear localization. It has been reported that the association of HSF1 with HSP90 keeps HSF1 in an inactive state by inhibiting HSF1 oligomerization. Accordingly, the dissociation of HSP90 from HSF1 allows oligomerization and activation of HSF1^6^. As shown in Fig. 4D,E, expression of IER5 resulted in decreased association of HSP90 with HSF1 and increased HSF1 oligomerization. Furthermore, expression of wild-type IER5, but not mutant IER5, resulted in a greater accumulation of HSF1 in the nucleus (Fig. 4F). Finally, we examined DNA binding by HSF1 in cell extracts using biotin-tagged DNA probes containing an HSE and bound to streptavidin-coated beads. Binding of HSF1 to HSE was strongly increased in cells overexpressing IER5 (Fig. 4G). These data collectively indicate that IER5 induces HSF1 activation and transactivation of the *HSP* family of genes.

### IER5 dephosphorylates HSF1 at multiple Ser and Thr residues, including sites involved in the repression of HSF1 activity

**Figure 5:**
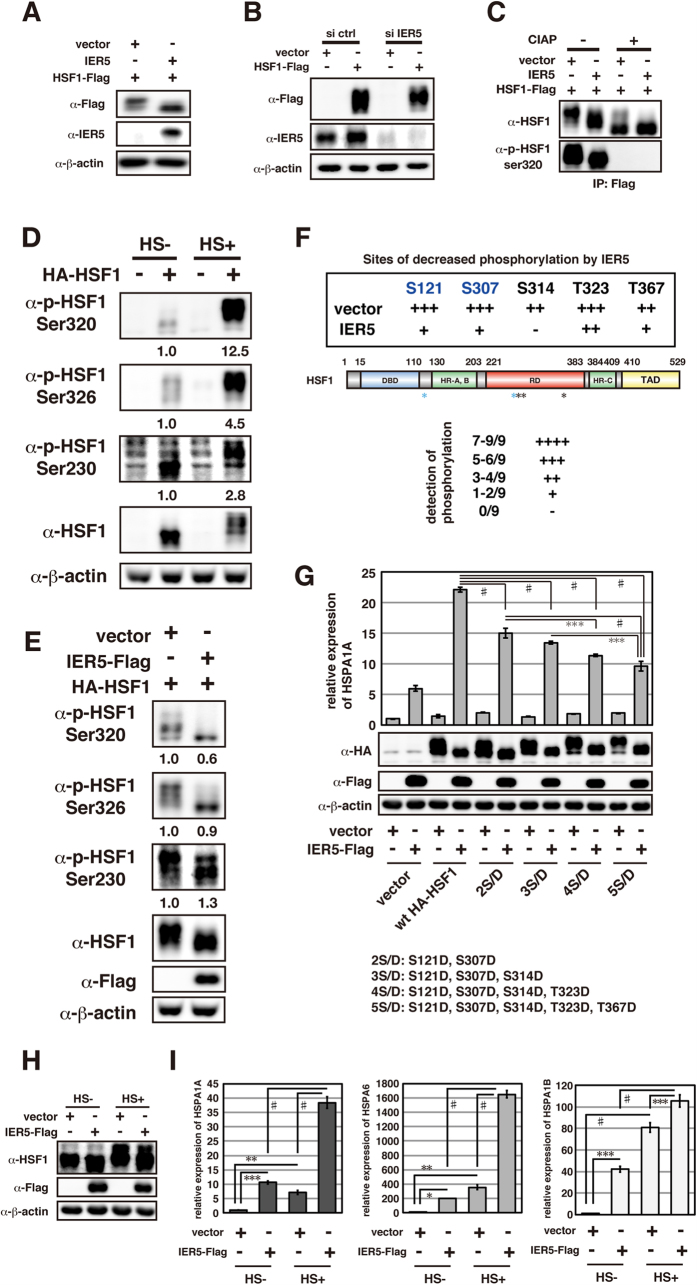
IER5 dephosphorylates HSF1 at multiple Ser and Thr residues including sites involved in repression of HSF1 activity. (**A–E**) Whole cell extracts and immunoprecipitated samples were analyzed by Western blotting. (**A**) 293T cells were transfected with HSF1-Flag together with control vector or an IER5 expression vector. Cells were harvested 21 hrs post-transfection. (**B**) 293T cells were transfected with control vector or HSF1-Flag expression vector, together with control or IER5-targetting siRNAs. Cells were harvested 49 hrs post-transfection. (**C**) 293T cells were transfected with HSF1-Flag and control vector or IER5. Cells were harvested 27 hrs post-transfection. Cell lysates were immunoprecipitated using anti-Flag antibody, and incubated with or without CIAP for 30 min. Total HSF1 and p-Ser320 were detected. (**D**) 293T cells were transfected with control vector or HA-HSF1 expression vector. Cells were treated with or without heat shock at 42 °C for 3 hrs, and harvested 24 hrs post-DNA transfection. (**E**) 293T cells were transfected with HA-HSF1 and control or IER5-Flag expression vector. Cells were harvested 24 hrs post-DNA transfection. (**F**) HSF1 modification was analyzed by LC-MS/MS. The experiment was performed nine times, and the numbers of analysis that detected each phosphorylation are shown. Significant reductions in phosphorylation at 5 residues (S121, S307, S314, T3232 and T367) were detected. Phosphorylation sites previously reported to be involved in the repression of HSF1 activity (S121 and S307) are shown in blue. (**G**) 293T cells were transfected with the indicated plasmids and harvested 24 hrs post-transfection. *HSPA1A* mRNA expression was analyzed by quantitative RT-PCR and other proteins were detected by Western Blotting. (***p < 0.001, ^#^p < 0.0001). (**H,I**) 293T cells were transfected with control vector or IER5-Flag expression vector. Cells were treated with or without heat shock at 42 °C for 3 hrs, and harvested 24 hrs post-DNA transfection. Endogenous HSF1, IER5-Flag protein expression was analyzed by Western blotting (**H**), and *HSPA1A*, *HSPA6* and *HSPA1B* mRNA expression was analyzed by quantitative RT-PCR (**I**). (*p < 0.05, **p < 0.01, ***p < 0.001, ^#^p < 0.0001).

HSF1 activation is regulated by various post-translational modifications, especially phosphorylation. For example, phosphorylation at Ser 230, 320 and 326 has been reported to increase HSF1 transactivation^20^,^21^,^22^. We therefore asked if HSF1 phosphorylation is affected by expression of IER5. We observed that HSF1 shifts to a higher electrophoretic mobility in cells expressing IER5 (Fig. 5A). Conversely, ablation of endogenous IER5 expression resulted in decreased HSF1 electrophoretic mobility (Fig. 5B). When HSF1 protein was treated with phosphatase prior to electrophoresis, no difference in HSF1 migration between IER5-expressing and control cells was observed (Fig. 5C). These results show that IER5 dramatically changes the post-translational modification of HSF1, in particular causing HSF1 dephosphorylation. Heat shock, which activates HSF1, induced strong phosphorylation of serines known to be involved in HSF1 activation: Ser230, 320 and 326 (Fig. 5D). In contrast, HSF1 phosphorylation at these sites was relatively unaffected by IER5 expression (Fig. 5E).

In order to comprehensively identify the modifications induced by IER5, we analyzed HSF1 in IER5-expressing versus control cell extracts by LC-MS/MS. We found that expression of IER5 was associated with decreased phosphorylation levels at multiple sites (Fig. 5F). In particular, significant reductions in phosphorylation were observed at 5 residues (S121, S307, S314, T323 and T367). We also observed a reduction in the acetylation of E113 and K118 (Fig. S3). Interestingly, the amino acids exhibiting these modification differences reside within the regulatory domain (RD, a.a. 221–383) or the flexible linker region (a.a. 110–130) of HSF1. The RD is located between the heptad repeat (HR)-A/B and the HR-C domain, and is involved in the repression of HSF1 activity. It has been reported that various post-translational modifications in the RD modulate its ability to repress HSF1 function. For example, phosphorylation of Ser320 and 326 increases HSF1 activity, while phosphorylation of Ser303 and 307 represses HSF1 activity^23^,^24^,^25^. In addition, the flexible linker region that connects the HSF1 DNA binding domain and the HR-A/B domain has been reported to be involved in the regulation of HSF1 activity and to influence HSF1 trimerization^6^. For example, phosphorylation of Ser121 within the linker region promotes HSP90 binding, which in turn represses HSF1 trimerization^26^. IER5 expression significantly reduces phosphorylation of Ser121 and 307, which are involved in the negative regulation of HSF1 activity (Fig. 5F). We therefore next analyzed whether HSF1 activation by IER5 involves the dephosphorylation of S121, S307, S314, T323 and/or T367. As shown in Fig. 5G, endogenous HSF1 induced expression of *HSPA1A*, an indicator of HSF1 activation, in a manner dependent on IER5. *HSPA1A* was not induced by ectopic expression of HSF1 alone, but was strongly induced when IER5 was expressed together with HSF1. We next constructed several mutant versions of HSF1 containing phosphorylation mimic mutants of these sites, as shown in Fig. 5G. We observed that IER5-dependent *HSPA1A* induction was significantly lower than wild-type when any of these mutants (2S/D, 3S/D, 4S/D and 5S/D) was expressed (Fig. 5G). In particular, we note that the 5S/D mutant dramatically reduces *HSPA1A* induction (22% activity compared to ectopic wild-type HSF1). These results suggest that dephosphorylation of S121, S307, S314, S323 and T367 are essential for HSF1 activation.

IER5 converts HSF1 to a hypo-phosphorylated active form, while heat shock converts HSF1 to a hyper-phosphorylated active form. To investigate whether IER5-mediated dephosphorylation of HSF1 can further activate HSF1 under conditions of heat stress, we subjected control or IER5-expressing cells to heat shock treatment (Fig. 5H,I). Induction of *HSP* family genes following heat shock was normal in control cells, but dramatically enhanced in cells expressing IER5. An increase in HSF1 electrophoretic mobility was also observed in heat-shocked cells expressing IER5 compared to HSF1 in heat-shocked cells in which IER5 was not expressed (Fig. 5H). Dephosphorylation of HSF1 by IER5 can therefore further activate HSF1 above the levels of activation induced by heat shock alone.

### IER5 regulates dephosphorylation of HSF1 by PP2A

**Figure 6:**
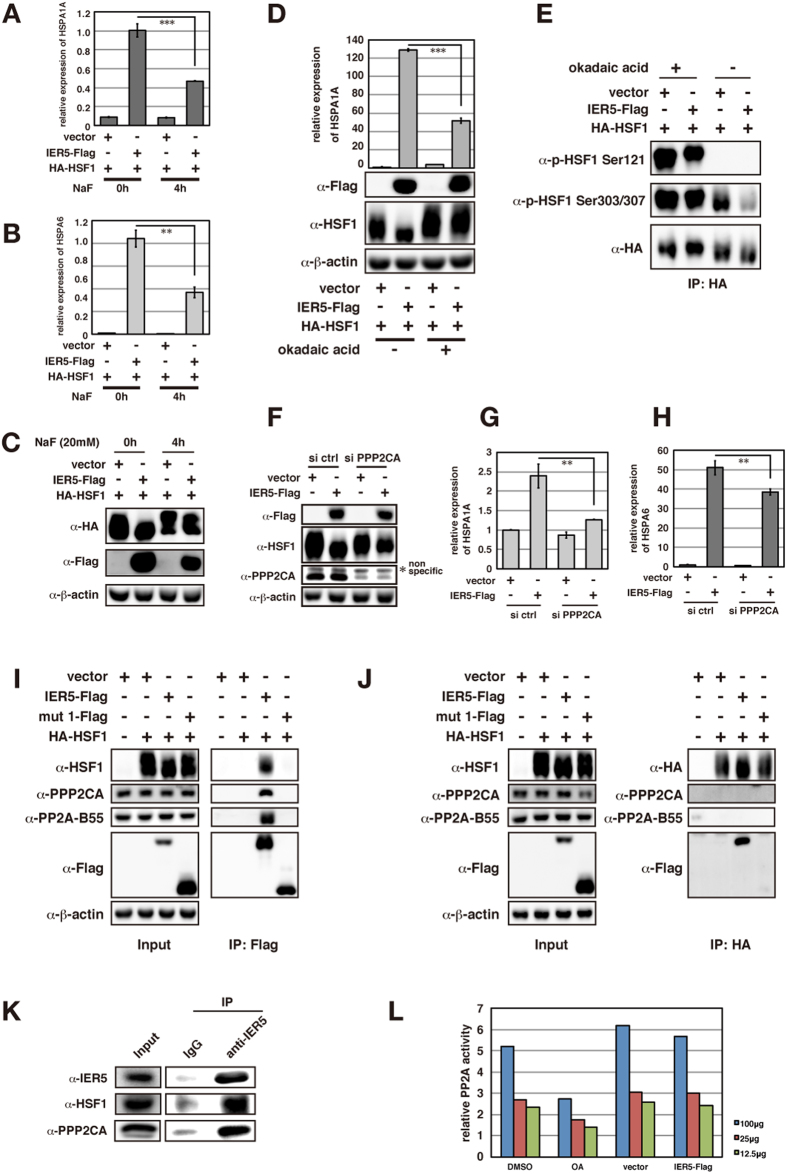
IER5 induces PP2A-dependent dephosphorylation and activation of HSF1. (**A–C**) 293T cells were transfected with HA-HSF1 together with control vector or IER5-Flag. Cells were treated with 20 mM NaF acid for 4 hrs and harvested 24 hrs post-transfection. Expression of *HSPA1A* and *HSPA6* were analyzed by quantitative RT-PCR (**A,B**) and expression of IER5-Flag and HA-HSF1 were detected by Western Blotting (**C**). (**p < 0.01, ***p < 0.001). (**D**) 293T cells were transfected with HA-HSF1 together with control vector or IER5-Flag. Cells were treated with 500 nM okadaic acid for 8 hrs and harvested 24 hrs post-transfection. Expression of *HSPA1A* was analyzed by quantitative RT-PCR and expression of IER5-Flag and HA-HSF1 were detected by Western Blotting. (***p < 0.001). (**E**) 293T cells were transfected as in (**D**). Cells were treated with 250 nM okadaic acid for 4 hrs and harvested 24 hrs post-transfection. Cell lysates were immunoprecipitated using anti-HA antibody. Total HSF1 and HSF1 phosphorylated at Ser121 or p-Ser303, 307 were detected by Western Blotting. (**F–H**) Control or PPP2CA (PP2A catalytic subunit)-targeting siRNAs were introduced into 293T cells. Subsequently, cells were transfected with HA-HSF1 and control vector or IER5-Flag expression vector 72 hrs post-siRNA transfection. Cells were harvested 24 hrs post-DNA transfection. IER5, HSF1 and PPP2CA protein levels were analyzed by Western blotting, and *HSPA1A* (**G**) and *HSPA6* (**H**) mRNAs were analyzed by quantitative RT-PCR. (**p < 0.01). (**I,J**) 293T cells were transfected with HA-HSF1 together with control vector, IER5-Flag or IER5-Flag mut 1-Flag, and harvested 24 hrs post-transfection. IER5-Flag (**I**) or HA-HSF1 (**J**) was immunoprecipitated using anti-Flag or anti-HA antibodies. Association of HSF1, PP2A catalytic subunit PPP2CA and PP2A regulatory subunit B55 with IER5 was analyzed by Western blotting in (**I**), and association of IER5, PPP2CA and B55 with HSF1 was analyzed in (**J**). (**K**) Endogenous IER5 in OE33 cells was immunoprecipitated using anti-IER5 antibody. Association of HSF1 and PPP2CA with IER5 was analyzed. (**L**) PP2A phosphatase activities were analyzed using the indicated amounts of cell lysates. 293T cells were treated with or without Okadaic acid (500 nM) for 8 hrs. Control or IER5-Flag expression vector were transfected and harvested 24 hrs post-transfection.

Our results indicate that IER5 modulates HSF1 activity by inducing dephosphorylation at serine and threonine residues, suggesting the potential involvement of a Ser/Thr protein phosphatase. To test this idea, we treated the cells with NaF, a general inhibitor of Ser/Thr protein phosphatases, and found that IER5-activated, HSF1-dependent induction of two *HSP* family genes was significantly reduced (Fig. 6A,B). Moreover, NaF treatment caused HSF1 to be hyper-phosphorylated, indicating the involvement of a Ser/Thr protein phosphatase that constitutively dephosphorylates HSF1 (Fig. 6C). One potential candidate involved in this response is PP2A, a regulatory subunit of which was reported to associate with IER5, and which was reported to interact with the HSF family protein HSF2^27^,^28^. PP2A is a phosphatase involved in various biological phenomena, including tumorigenesis^29^,^30^. To examine whether PP2A is the phosphatase responsible for the dephosphorylation and activation of HSF1 by IER5, we treated cells expressing IER5 and HSF1 with okadaic acid, an inhibitor of PP2A. As shown in Fig. 6D, okadaic acid treatment resulted in the decreased electrophoretic mobility of HSF1 and reduced induction of *HSPA1A*. Okadaic acid also dramatically enhanced the phosphorylation of Ser121 and 303/307, suggesting that PP2A may dephosphorylate these residues (Fig. 6E). Lastly, we knocked down PP2A expression in IER5-expressing cells and found that this caused a decrease in HSF1 electrophoretic mobility, as well as a decrease in *HSPA1A* and *HSPA6* induction (Fig. 6F–H). These results collectively indicate that PP2A is required for HSF1 activation and HSF1 dephosphorylation by IER5.

### IER5 forms a ternary complex with HSF1 and PP2A

We next analyzed the mechanism by which IER5 and PP2A activate HSF1. As shown before in Fig. 4F, expression of IER5 results in HSF1 accumulation in the nucleus and co-localization with IER5, suggesting a possible physical association of these two factors. To test this, we transfected 293T cells with Flag-tagged wild-type- or mutant IER5 and HA-tagged HSF1. Next, IER5 and HSF1 were immunoprecipitated with anti-Flag or anti-HA antibody, respectively. Figure 6I,J show that HSF1 associates with wild-type IER5, but not with mut 1, a mutant of IER5 incapable of activating HSF1. In addition, we observed that IER5 was associated with the PP2A catalytic subunit PPP2CA and regulatory subunit B55 (Fig. 6I), confirming a previous study that comprehensively identified PP2A-interacting proteins^27^. Similar associations were observed between endogenous IER5 and HSF1, and between endogenous IER5 and PP2A (Fig. 6K). We could not detect an association of PPP2CA with HSF1 under the conditions employed (Fig. 6J). Since HSF1 is a substrate of PP2A, it is possible that PP2A dissociates from HSF1 when HSF1 is dephosphorylated, and therefore this association may be difficult to detect. Furthermore, PP2A phosphatase activity was not affected by IER5 expression (Fig. 6L). Collectively, these results suggest that IER5 functions as a scaffold to bring HSF1 and PP2A in proximity to one another, thereby allowing PP2A to dephosphorylate and activate HSF1.

### The IER5/HSF1 axis is required for cancer cell proliferation

**Figure 7:**
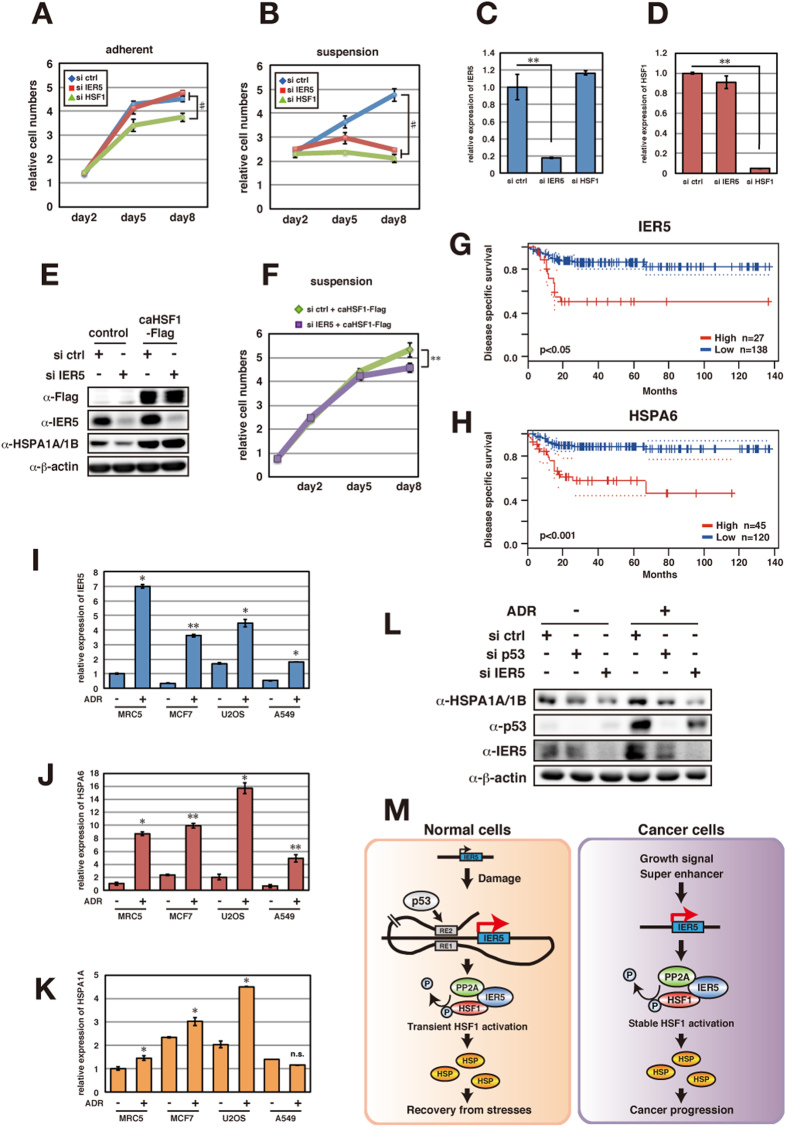
IER5/HSF1/HSP family gene axis in cancer and in stressed cells. (**A–D**) OE33 cells (2 × 10^3^ cells) were plated in adherent (**A**) or suspension (**B**) 96 well culture plates, and control, IER5-targeting or HSF1-targeting siRNAs were introduced. Cell growth assays were performed on the indicated days. Relative cell numbers were analyzed with CellTiter-Glo reagents from four wells and the mean cell numbers ±SD are shown. *IER5* and *HSF1* mRNA expression was analyzed by quantitative RT-PCR 48 hrs post-transfection (**C,D**). (**p < 0.01, ^#^p < 0.0001). (**E**) OE33 cells were stably transfected with caHSF1, and the indicated siRNAs were introduced. Cell lysates were prepared 48 hrs post-transfection, and expression of caHSF1-Flag, IER5, HSPA1A/1B were analyzed by Western blotting. (**F**) Cell growth assays were performed as in (**B**) using OE33 cells expressing caHSF1. (**p < 0.01). (**G,H**) Expression of *IER5* (**G**) and *HSPA6* (**H**) and prognosis in cancer patients. Disease-specific survival of patients with bladder cancer (Transitional cell carcinoma, dataset GSE13507) was analyzed using the PrognoScan database. (**I–K**) Expression of *IER5* (**I**), *HSPA6* (**J**) and *HSPA1A* (**K**) mRNA in cells treated with Adriamycin. Cell lines carrying wild-type p53 were treated with Adriamycin (1 μM) for 24 hrs (MRC5, MCF7, U2OS) or 19 hrs (A549). (*p < 0.05, **p < 0.01). (**L**) Expression of IER5, p53 and HSPA1A protein level in U2OS cells. Control, p53 and IER5 targeting siRNAs were introduced. Cells were treated with Adriamycin (1 μM) for 24 hrs and harvested 48 hrs post siRNA-transfection. (**M**) IER5 is transiently induced downstream of p53 and activates HSF1 in stressed cells, while IER5 is overexpressed and constitutively activates HSF1 in cancer cells.

We have shown that IER5 is required for the anchorage-independent growth of cancer cells (Fig. 2) and is an activator of HSF1. We speculate that IER5-mediated HSF1 activation is especially required for the protection of cells under stress, such as that which occurs when cells are placed in suspension cultures. We therefore next examined the potential involvement of HSF1 in anchorage-independent cell growth. As shown in Fig. 7A,B, knockdown of HSF1 suppressed cell growth similar to knockdown of IER5 in OE33 cells. Growth suppression was minor for adherent cells, but was significant for cells growing under conditions of stress caused by loss of anchorage. We confirmed that *IER5* and *HSF1* were indeed depleted under these experimental conditions (Fig. 7C,D). Knockdown of HSF1 or IER5 in HeLa cells caused similar suppression of cell proliferation (Fig. S4). Furthermore, we took a constitutively active form of HSF1 (caHSF1), which has the ability to induce HSF1 target genes independently of HSF1 activating signal^31^ and expressed this in OE33 cells. These cells no longer required IER5 for HSP family expression, and HSP family proteins were constitutively expressed regardless of IER5 status (Fig. 7E). Subsequently, we treated caHSF1-expressing cells with siRNA targeting IER5 in addition to the parental OE33 cells. While the parental OE33 cells required IER5 for proliferation from day 5 (Fig. 7B), caHSF1 expression rescued the proliferation of cells in which IER5 is knocked down completely up to day 5 (Fig. 7F). Theses results show that HSF1 activation downstream of IER5 is important for proliferation of cancer cells.

### IER5 is responsible for HSF1 activation in cancers and high expression of IER5 is associated with poor prognosis of cancer patients

Search of a publicly available cancer microarray database (PrognoScan;^32^) revealed that higher *IER5* expression is associated with poorer prognosis in bladder, breast and brain cancer patients (Fig. 7G and S5). Interestingly, higher *HSPA6* (the gene most strongly induced by IER5 expression, shown in Fig. 3A) expression is also related to poorer prognosis, and we observed overall similar patterns of survival among bladder and brain cancer patients exhibiting high versus low *IER5* and *HSPA6* expression (Fig. 7H and S5). The correlation between *IER5* and *HSPA6* expression is also quite strong (Fig. 7G and S5, r = 0.47 and p < 0.0001 in bladder cancer samples, r = 0.63 and p < 0.0001 in breast cancer samples and r = 0.2896 and p < 0.005 in brain cancer samples). These results suggest that the IER5-HSF1-HSP family axis of regulation may be involved in cancer progression. The association of *IER5* and *HSPA6* expression with cancer progression may be related to the *in vitro* observation that IER5 and HSF1 are required for the proliferation of cancer cells under stressed conditions.

It has been reported that when HSF1 is activated in cancers, it specifically induces expression of cancer-associated HSF1 target genes in addition to the HSP family of genes^8^. We therefore analyzed the correlation between *IER5* mRNA levels and the expression of cancer-associated HSF1 target genes in cancer patients. As shown in Fig. S5A, expression of *IER5* and cancer-associated HSF1 target genes (*RBM23* and *EIF4A2*) showed a significant association in bladder cancer (Fig. S5A, r = 0.64 and p < 0.0001 for *RBM23*, and r = 0.54 and p < 0.0001 for *EIF4A2*). In addition, an even higher association of *IER5* and *RBM23* and *EIF4A2* were found in breast cancer patients (Fig. S5B, r = 0.75 and p < 0.0001 for *RBM23*, and r = 0.65 and p < 0.0001 for *EIF4A2*). These results collectively indicate that IER5 is responsible for HSF1 activation in cancer.

### The *HSP* family of genes is induced by DNA damage in a p53- and IER5-dependent manner

As described earlier, *IER5* gene is a target of p53 and its expression is induced under conditions of cellular stresses. Therefore, we examined if *HSP* family gene expression can be induced by DNA damage. As shown in Fig. 7I–K, Adriamycin treatment resulted in the concomitant upregulation of *IER5, HSPA6* and *HSPA1A* mRNAs in several cell lines containing wild-type p53. In addition, HSPA1A protein upregulation by Adriamycin treatment was dependent on both p53 and IER5 (Fig. 7L). Thus, our data show that HSP family proteins are induced by p53 and IER5 upon DNA damage, and may play a role in the recovery and protection of normal cells, as well as of cancer cells having wild-type p53.

## Discussion

### p53-dependent and -independent *IER5* gene induction

We previously showed by microarray expression analysis that *IER5* is a p53-inducible gene^10^, and we now have shown by ChIP-seq analysis that the *IER5* is directly regulated by p53. We also have shown that the RE2 motif, a p53 binding site 46 kb downstream of the *IER5* gene, has all the properties of an active enhancer, and can function as an enhancer of the *IER5* gene. Recently, Melo *et al.* performed a comprehensive genome-wide analysis of p53 binding sites, and reported that the *IER5* gene is regulated by p53^33^. They further reported that the *IER5* promoter region interacts intrachromosomally with the same region that we identified as RE2. We have shown that there is a weak, but detectable *IER5* promoter-RE2 interaction under non-stressed conditions. This interaction was weaker but still detectable in HCT116 *p53*−/− cells. We also have shown that *IER5* is expressed in a p53-independent manner, and that the region containing the RE2 motif functions as a super-enhancer in various cancer cell lines, regardless of p53 functional status. It will be interesting to analyze whether this *IER5* promoter-RE2 interaction is required for p53-independent *IER5* gene expression in such cell lines. We would also like to clarify which transcription factors are involved in the upregulation of *IER5* mRNA in cancer.

### A novel mechanism of HSF1 activation by IER5

The activation and extensive phosphorylation of HSF1 following heat stress has been well characterized^6^. Phosphorylation occurs on multiple residues, including Ser230, Ser320 and Ser326, and these enhance the transcriptional activity of HSF1^20^,^21^,^22^. In this study, we showed that IER5 expression activates HSF1, but this activation involves HSF1 hypo-phosphorylation at multiple residues including Ser121, Ser307, Ser314, Thr323 and Thr367. In addition to the previously known HSF1 phosphorylation sites that are involved in HSF1 inactivation (Ser121 and Ser307^23^,^24^,^25^,^26^), Ser314, Thr323 and Thr367 are novel sites that were identified in this study. We observed that IER5 forms a ternary complex with HSF1 and PP2A to dephosphorylate and activate HSF1. PP2A is a ubiquitously expressed protein that dephosphorylates many different intracellular targets^29^,^30^. PP2A incorporates 15 unique B-regulatory subunits, and it has been demonstrated that these B subunits control the substrate specificity and localization of the PP2A holoenzyme. Since it has been reported that IER5 associates with one of the B subunits of PP2A, PPP2R2B, we speculate that induction of IER5 may alter the substrate specificity of PP2A via interaction with one or more of its subunits and thereby promote the dephosphorylation and consequent activation of HSF1^27^.

Very recently, Ishikawa *et al.* reported that there is a functional connection between HSF1 and IER5 under heat stress conditions^34^,^35^. They have identified *IER5* as a target gene of HSF1 upon heat stress, and reported that IER5 forms a complex with HSF1 and the PP2A regulatory subunit B55^34^,^35^. Their data is similar to our results shown in Fig. 5J,I, and our data also confirms the importance of IER5 in the response to heat shock stress. In this manuscript, we have further shown that the IER5-mediated HSF1 activation can also occur in the absence of heat stress, and is highly important for proliferation of cancer cells. Our results provide several novel insights into HSF1 function, showing IER5 facilitates the generation of a novel hypo-phosphorylated form of HSF1 that is active, yet is distinct from the hyper-phosphorylated form induced by heat shock, and involves distinct sites of phosphorylation. While the mechanisms of HSF1 activation in response to heat stress have been well studied, HSF1 activation in response to other signals has not been well understood to date. Thus, our study provides a novel example of HSF1 activation by a signal other than heat stress.

### HSF1 activation in cancer requires IER5

Recent findings have highlighted the potential importance of HSF1 activation in cancer. It has been suggested that growth-stimulating signals such as those transduced via the MAPK pathway can activate HSF1^36^, although the precise mechanism by which this might occur is unknown. Because HSF1 activity is normally regulated by post-translational mechanisms, upregulation of *HSF1* mRNA or protein expression alone would not be expected to lead to enhanced HSF1 activity or tumorigenicity. Indeed, it has been reported that overexpression of HSF1 does not transform MEFs^9^. Conversely however, MEFs lacking HSF1 are refractory to transformation, indicating that HSF1 is critical for tumorigenesis^9^. We have shown that *IER5* expression is induced by growth stimuli, is overexpressed in various cancers, that the *IER5* gene is associated with super-enhancers in cancer cells and that high *IER5* expression is related to poorer prognosis of cancer patients. We also have shown that mRNA expression of IER5 and HSF1 target genes show high correlation in several cancers. In addition, in cancer cells that exhibit enhanced *IER5* expression, suppression of IER5 by siRNA results in the downregulation of HSF1 activity and cell proliferation. These results collectively indicate that IER5 is responsible for HSF1 activation in cancers and supports cancer cell proliferation.

### The p53-IER5-HSF1 axis in normal and cancer cells

In this report, we have mainly focused on the tumor-promoting functions of IER5. However, HSF1 and the *HSP* family of genes are well known to regulate cellular homeostasis and promote the survival of normal cells^37^. Therefore the function of IER5 downstream of p53 in normal cells is presumably to elicit a recovery and survival response under conditions of stress by activating HSF1 and upregulating the *HSP* family of genes downstream of HSF1 (Fig. 7M).

It is well known that approximately half of all human cancers carry a p53 mutation, illustrating the importance of p53 function in tumor suppression. However, it is of note that the remaining half retains wild-type p53. Indeed, we imagine that some cancer cells may positively select for the retention of wild-type p53. As we have shown, the *IER5* gene is associated with super enhancers in cancers, indicating that it is a key gene involved in tumor pathogenesis. Therefore, some cancer cells may select for wild-type p53 because induction of IER5 (and downstream activation of HSF1) by p53 can protect the cells under conditions of stress resulting from oncogenic transformation, such as lack of anchorage or DNA damage. The role of the p53-IER5-HSF1 axis in the maintenance of cellular homeostasis in normal cells and its contribution to tumorigenesis *in vivo*, particularly, the function of IER5 in tumors containing wild-type p53, is an important future issue, which we would like to address using *IER5*-deficient mice.

## Methods

### Northern blotting analysis and microarray expression analysis

RNA was prepared using an RNeasy Mini kit (QIAGEN). Northern blotting was performed as described^10^. Methylene blue staining of 28S ribosomal RNA confirmed equal loading of RNA in each lane. Probes were prepared using a BcaBEST labeling kit (TaKaRa), and purified by serial purification using a Probe Quant G-50 MicroColumn (Amersham) and NICK Column (Amersham). The full open reading frames of genes obtained from EST clones, purchased from Open Biosystems, were used for probe preparation. The EST clones were IER5 (IMAGE ID 3457186), HSP70 (IMAGE ID 3345864), HSPA6 (IMAGE ID 5723718) and DNAJB1 (IMAGE ID 2821008). Microarray expression analysis was performed as described^10^.

### Western blotting analysis

Cells were lysed in lysis buffer containing 50 mM Tris-HCl (pH 8.0), 1% NP40, 250 mM NaCl, 50 mM NaF, 1 mM Na_3_VO_4_, 1 mM protease inhibitor (PMSF, aprotinin, leupeptin) and 1 mM DTT. Whole cell lysates were subjected to protein quantification and analyzed by Western blotting. Antibodies used in this study: anti-IER5 rabbit polyclonal antibody (HPA029894), anti-Flag M2 mouse monoclonal antibody (F1804), anti-β-actin mouse monoclonal antibody (A2228) from SIGMA, anti-p53 goat polyclonal antibody (sc-6243-G), anti-p21 rabbit polyclonal antibody (C-19), anti-HSF1 rabbit polyclonal antibody (sc-9144), anti-phospho-HSF1 (Ser230) rabbit polyclonal antibody (sc-30443-R), anti-PP2A-B55 (sc-18330) from Santa Cruz Biotechnology, anti-phospho-HSF1 (Ser320) rabbit monoclonal antibody (#2446-1) and anti-phospho-HSF1 (Ser326) rabbit monoclonal antibody (#2092-1) from Epitomics, anti-phospho HSF1 (Ser121) rabbit polyclonal antibody from Assay bio Tech, anti-phospho-HSF1 (Ser303/Ser307) rabbit monoclonal antibody (ab81281) from abcam, anti-HSPB1 mouse monoclonal antibody (ADI-SPA-800), anti-DNAJB1 rabbit polyclonal antibody (ADI-SPA-400), anti-HSPA6 mouse monoclonal antibody (ADI-SPA-754), anti-HSPA1A/1B mouse monoclonal antibody (ADI-SPA-810) from Enzo Life Sciences, anti-HA mouse monoclonal antibody (12CA5) from Roche, and anti-PP2A C subunit (clone 1D6) from Merck Millipore.

### Immunoprecipitation

Lysis buffer used for each experiment is shown below.

Figures 4D and 6I–L: 50 mM HEPES (pH 7.0), 150 mM NaCl, 1 mM EDTA, 2.5 mM EGTA, 10% glycerol, 0.1% NP-40, 0.1 mM Na_3_VO_4_, 1 mM NaF, 0.1 mM DTT and a protease inhibitor cocktail.

Figures 5C–E and 6E: 50 mM Tris-HCl (pH 8.0), 1% NP40, 250 mM NaCl, 50 mM NaF, 1 mM Na_3_VO_4_, 1 mM protease inhibitor (PMSF, aprotinin, leupeptin) and 1 mM DTT.

For immunoprecipitation of Flag-tagged and HA-tagged proteins, cell lysates were immunoprecipitated with M2-agarose beads (SIGMA) or EZview Red Anti-HA Affinity Gel (SIGMA). For immunoprecipitation of IER5, anti-IER5 rabbit polyclonal antibody (HPA029894) and Protein A beads (GE Healthcare) were used.

### Plasmids

IER5 and HSF1 expression constructs: the IER5 and HSF1 cDNAs were each cloned into the pcDNA3 vector as described^10^. Constitutively active HSF1 was constructed by deleting amino acids 186–202 in the second leucine zipper of HSF1^31^.

Plasmids carrying one or two copies of wild-type or mutant p53RE (p53RE1, p53RE2, p53RE2 mut): Plasmids were obtained by cloning double-stranded oligonucleotides into the pGL3-promoter vector (Promega), which contains a minimal promoter. Oligonucleotide sequences: p53RE1 (5′-AAACAGGTTGAGACATGTCC-3′), p53RE2 (5′-AGGCATGCCCGGGCATGTCT-3′), p53RE2 mut (5′-AGGAATTCCCGGGAATTTCT-3′).

HSPA1A, HSPA1B, HSPA6 promoter-reporter constructs:

The 1kb region upstream of the transcription start site of each gene was amplified by PCR, and cloned into the Xho I/Bgl II site of the pGL3-promoter vector (Promega).

Primers used to amplify promoter regions:

HSPA1A.

5′ primer : 5′-TTAGTCGAGAAAAAAAAAAATTAAAAATAAATAA-3′.

3′ primer : 5′-TTAAGATCTGGCCATCCGGTTCCCTGCTCTCTGT-3′.

HSPA1B.

5′ primer : 5′-TTAGTCGAGCTAAAAACGGTAACAGCCTAGGGGT -3′.

3′ primer : 5′-TTAAGATCTCATCCGGTGCCCTGCTGCTGTGGGC -3′.

HSPA6.

5′ primer : 5′-TTAGTCGAGAACCTTCAGAAGTCTCAGAGAAATG -3′.

3′ primer : 5′-TTAAGATCTCATGGCTGAAGCTTCTTGTCGGATG -3′.

HSPA1A 1–4 promoter-reporter constructs:

The corresponding regions were amplified by PCR from human genomic DNA, and cloned into the Xho I/Bgl II site of the pGL3-promoter vector.

Primers used to amplify promoter regions:

Promoter 1.

5′ primer : 5′-TTAGTCGAGCCCCCGCCTCCCCCATTGTGGCTGC -3′.

Promoter 2.

5′ primer : 5′-TTAGTCGAGCCCTGCAAATTTGAGACGGCTCCAA -3′.

Promoter 3.

5′ primer : 5′-TTAGTCGAGACAATTAAAAGCCCAGCGCCGACCC -3′.

Promoter 4.

5′ primer : 5′-TTAGTCGAGCAGGACGGGAGGCGAAAACCCTGGA -3′.

3′ primer (commonly used for all constructs);

5′-TTAAGATCTGGCCATCCGGTTCCCTGCTCTCTGT-3′

### ChIP sequence

ChIP assays were performed as described^38^. For p53 induction, cells were treated with 5-FU (0.375 mM for 9 hrs). Antibodies against p53 (FL393, Santa Cruz), H3K27ac, H3K4me1, H3K4me3 (07–473, Millipore) or phospho-RNAP II were used to precipitate immune complexes. Antibodies against H3K27ac, H3K4me1 and phospho-RNAP II were kindly provided by Dr. Hiroshi Kimura, Tokyo Institute of Technology. DNA co-precipitating with p53, H3K27ac, H3K4me1, H3K4me3 or phospho-RNAP II (5–10 ng) was purified by SDS–PAGE to obtain 100–300 bp fragments and sequenced on an Illumina 1G sequencer. Size-fractionated DNA was extracted and a single adenosine was added using Klenow exo– (3′ and 5′ exo minus; Illumina). Illumina adaptors were then added and DNA was subjected to 20 cycles of PCR according to manufacturer’s instructions. We then purified the DNA and performed cluster generation and 36 cycles of sequencing on the Illumina cluster station and 1G analyzer following the manufacturer’s instructions. The resulting sequences were mapped to the build #36 reference human genome. ChIP signal values for p53, k4me3 (5-FU 0, 9h), K4me1 (5-FU 0, 9h), K27ac (5-FU 0, 9h) and Pol2 (5-FU 0, 9h) were 26730379, 17020836, 22559531, 8296440, 9319944, 29174994, 27258981, 15964489 and 15142757, respectively. ChIP signal values (ChIP counts/estimated or input counts) were generated as previously reported^39^. Probability values were generated as all fragments were randomly mapped to the non-repetitive sequences of the Human Genome.

### Circularized Chromosome Conformation Capture (4C)

4C-seq assays were performed as previously reported^40^,^41^. Chromatin from 10-million cells was cross-linked with 1% formaldehyde for 10 min at room temperature, then cells were treated with lysis buffer (50 mM Tris-HCl, 150 mM NaCl, 5 mM EDTA, 0.5% IGEPAL CA-630 (Sigma-Aldrich, I8896), 1% Triton X-100). Nuclei were digested with NlaIII (NEB, R0125L) and ligated with T4 DNA ligase (Roche, 799009). A secondary restriction digestion was performed with DpnII (NEB, R0543M), followed again by ligation. Specific 4C primers were designed near a p53 binding site. Illumina adaptor sequence and index sequence were included in the primer sequence. Sixteen-PCR reactions were performed with Expand Long Template PCR System (Roche, 759 060 001), pooled together and purified using Agencourt AMPure XP beads (Beckman, A63882).

### PCR primers for 4C-seq

Following primers were used for 4C-seq library preparation.

p53 RE2_Forward;

5′-AATGATACGGCGACCACCGAGATCTACACTCTTTCCCTACACGACGCTCTTCCGATCTTTTAAATGCCAGTGTCCATG-3′

p53 RE2_Reverse1;

5′-CAAGCAGAAGACGGCATACGAGATATCACGGTGACTGGAGTTCAGACGTGTGCTCTTCCGATCTTCTCTACAGAGAGCCAGCTT-3′

p53 RE2_Reverse2;

5′-CAAGCAGAAGACGGCATACGAGATTTAGGCGTGACTGGAGTTCAGACGTGTGCTCTTCCGATCTTCTCTACAGAGAGCCAGCTT-3′

p53 RE2_Reverse3;

5′-CAAGCAGAAGACGGCATACGAGATGATCAGGTGACTGGAGTTCAGACGTGTGCTCTTCCGATCTTCTCTACAGAGAGCCAGCTT-3′

### Reverse transcription and real-time PCR

Reverse transcription was carried out using the SuperScript First-Strand Synthesis System for RT-PCR (Life Technologies) or ReverTra Ace (TOYOBO) following the manufacturer’s instructions. Total RNA (0.2–5 μg) was used for reverse transcription. Reverse-transcribed cDNAs were subjected to real-time PCR, which was performed with a LightCycler 480 Instrument (Roche Diagnostics) or CFX96 Touch Real-Time PCR System (Bio-Rad). For the detection of IER5, HSF1, HSPA1A and GAPDH, a TaqMan probe (IER5; Hs00275419, HSF1; Hs00232134_m1, HSPA1A; Hs00359163_s1, GAPDH; Hs02758991_g1)from Applied Biosystems were used. For the detection of HSPA6 and HSPA1B, a TaqMan probe from Integrated DNA Technologies was used. In some cases, custom-designed TaqMan Dual-Labeled Probes from Sigma-Aldrich were also used for the detection of HSPA1A and GAPDH.

### Transfection and luciferase reporter assay

Transfection and luciferase reporter assays were performed as described (10). Transient transfection assays were performed using Lipofectamine Plus reagent (Life Technologies). For the luciferase reporter assay shown in Fig. 1H, Saos2 cells were seeded in 96-well dishes and co-transfected with 60 ng of a firefly luciferase reporter gene and 3 ng of each p53 gene cloned in the pcDNA3 vector together with 15 ng of a Renilla luciferase expression vector (pGL4 [TK-Rluc] vector, Promega) as an internal control for transfection efficiency. For the luciferase reporter assay shown in Fig. 3, H1299 cells were co-transfected with 30 ng of a firefly luciferase reporter gene and 5 ng of each IER5 gene cloned in pcDNA3 vector together with 3 ng of a Renilla luciferase expression vector pGL4 [TK-Rluc] vector. Cells were harvested 48 hrs (Fig. 1H) or 24 hrs (Fig. 3) post-transfection, and analyzed using the Dual-Luciferase Reporter Assay System (Promega). All of the luciferase reporter assay data are the mean-fold activation +/− SD of three independent experiments. The siRNAs were introduced using RNAiMAX (Invitrogen, Carlsbad, CA). Control, ON-target plus IER5-targeting, HSF1-targeting, p53-targeting and PPP2CA-targeting siRNAs were purchased from Dharmacon Research (Lafayette, Colorado).

### Analysis of HSF1 modification by liquid chromatography/mass spectrometry/mass spectrometry (LC-MS/MS)

Identification of HSF1 modification was performed using LC-MS/MS. 293T cells were transfected with Flag-tagged HSF1 together with IER5 or control empty vector, and harvested 27 hrs post-transfection. Cells were lysed with lysis buffer containing 50 mM Tris-HCl (pH 8.0), 1% NP40, 250 mM NaCl, 50 mM NaF, 1 mM Na_3_VO_4_, 1 mM protease inhibitor (PMSF, aprotinin, leupeptin) and 1 mM DTT. For immunoprecipitation of Flag-tagged HSF1 protein, the lysate was immunoprecipitated with M2-agarose beads (SIGMA). The immunoprecipitated samples were separated by 10% sodium dodecyl sulfate-polyacrylamide gel electrophoresis (SDS-PAGE). Proteins were stained with SYPRO Ruby, excised and subjected to in-gel tryptic digestion with modified trypsin (Promega, Madison, WI) as described previously^42^. The tryptic digests were subjected to liquid chromatography (Paradigm MS4 dual solvent delivery system; Michrom BioResources, Auburn, CA) coupled with tandem mass spectrometry (LTQ Orbitrap XL; Thermo Fisher Scientific, San Jose, CA) equipped with a nanoelectrospray ion source (AMR, Tokyo, Japan). Mascot software (Matrix science, London, UK) was used to search for the mass of the peptide ion peaks against the SWISS-PROT database. The following search parameters were used: tolerance of 2 missed trypsin cleavages, variable modification on the methionine residue (oxidation, +16 Da), maximum precursor ion mass tolerance of ±10 ppm, and fragment ion mass tolerance of ±0.8 Da. Proteins with a Mascot score of 34 or more were considered as positively identified.

### Immunofluorescence

H1299 cells (6 × 10^4^ cells) were transfected with 320 ng of each expression vector plasmid, and harvested 24 hrs post-transfection. Cells were fixed with 4% paraformaldehyde in PBS for 10 min, and permeabilized with 0.2% Triton X-100 in PBST for 2 min. Cells were blocked with BSA (5 mg/ml) and 50 mM Glycine in PBST for 1 h. The cells were sequentially incubated with anti-Flag and anti-HSF1 (H-311) antibody for 1 h at room temperature and AlexaFluor 594 and 488-labeled secondary antibody (Molecular Probes, Eugene, OR, USA), and mounted with DAPI (2.5 μg/ml) in DABCO glycerol. Images were obtained by using a Keyence BZ-9000 fluorescence microscope.

### HSF1 crosslink

H1299 cells were transfected with an IER5 expression vector, harvested 25 h post-transfection and lysed in lysis buffer A containing 25 mM HEPES (pH 7.4), 0.5% Triton-X, 100 mM NaCl, 5 mM EDTA, 10 mM NaF, 1 mM Na_3_VO_4_, 1 mM protease inhibitor (PMSF, aprotinin, leupeptin) and 1 mM DTT. Whole cell lysates (2 × 10^6 ^cells/ml) were crosslinked with 0.1, 0.3 mM EGS [ethylene glycol bis] (Thermo SCIENTIFIC) at room temperature for 30 min. After quenching of crosslinking by addition of 50 mM Tris-HCl (pH 7.2), Western blotting was performed.

### HSF1 pull-down assay using biotinylated DNA probe

To analyze HSF1 binding to Heat Shock Elements (HSEs), we performed a streptavidin pull down assay. The HSE sequence from the HSPA1A gene was used (nt −132 to −109 of the HSPA1A gene; 5′-AAACCCCTGGAATATTCCCGACC-3′). A 2x repeat of the HSE element was cloned into the Pikka Gene basic vector (Toyo Ink Co.). Preparation of biotinylated DNA probes: HSE and empty vector were PCR amplified using biotinylated primers. The biotinylated probes were bound to streptavidin-coated beads (Magnesphere Paramagnetic Particles, Promega). Preparation of cell extracts: H1299 cells were transfected with an IER5-Flag expression vector, harvested 24 hrs post-transfection and lysed in lysis buffer A. To avoid non-specific binding, poly dI:dC (7 μg/ml) was added. Biotinylated DNA (1.5 μg) was bound to the magnetic beads and mixed with 1.9 mg of cell lysates. Incubation was performed for 7 hrs and beads were washed 5 times with wash buffer containing 20 mM Tris-HCl (pH 7.4), 0.1% Triton-X, 10% Glycerol, 1 mM EDTA, BSA (0.1 mg/ml). Bead-bound HSF1 was denatured in Laemmli buffer and analyzed by Western blotting.

### Cell growth assay

Cells were plated in 96 well adherent culture plates (Corning, 3610) or suspension culture plates (SUMILON, MS-8096R) and the indicated siRNAs were introduced into wells. Cell numbers were analyzed using CellTiter-Glo (Promega) in four or six wells and the mean cell numbers ± SD are shown.

### Colony formation assay in soft agar

Control or IER5-targeting siRNAs were introduced into cells. After 24 hrs, the cells (1 × 10^4 ^cells) were plated in a layer of 0.33% agarose/RPMI over a layer of 0.5% agarose/RPMI in a 3 cm dish and cultured for 9 days. Colonies with an image size larger than 600 square pixels were analyzed by ImageJ software and counted from four dishes and the mean numbers of colonies ±SD are shown.

### Measurement of PP2A phosphatase activity

PP2A phosphatase activities were analyzed in 293T cell lysates using a PP2A immunoprecipitation phosphatase assay kit (Millipore). Cell lysates were prepared by suspending the cells in lysis buffer (20 mM imidazole, 50 mM HEPES (pH 7.0), 150 mM NaCl, 1 mM EDTA, 2.5 mM EGTA and a protease inhibitor cocktail), followed by sonication (3 times, each 30 sec).

### Analysis of *IER5* and *HSPA6* expression using the Gene Logic SCIANTIS database and the PrognoScan database

*IER5* expression was analyzed using the Gene Logic SCIANTIS database (http://www.genelogic.com), a comprehensive genome-wide gene expression database annotated with detailed clinical and biological information. *IER5* mRNA expression levels were analyzed in kidney (renal cell carcinoma, clear cell type, primary), pancreas (adenocarcinoma, primary), ovary (adenocarcinoma, papillary serous type, primary), stomach (adenocarcinoma, primary), colon (adenoma), endometrium (adenocarcinoma, endometrioid type, primary), urinary bladder (transitional cell carcinoma, primary) cancer and their corresponding normal tissues.

Correlations between the expression of *IER5* and *HSPA6* and prognosis in cancer patients were analyzed using the PrognoScan database (http://www.abren.net/PrognoScan/), a large collection of publicly available cancer microarray data sets with clinical annotations and a tool for assessing the biological relationships between gene expression and prognosis.

### Statistical analysis

Data were calculated and shown as mean ± SD. Significance of differences was determined by Student *t* test (Figs 2A,F,G,I–L, 3D,E and 7F,I–K), one-way ANOVA (Figs 2D and 7A–D, S4A and S4B) or two-way ANOVA (Figs 4A,B, 5G,I and 6A,B,D,G,H). Correlation data were analyzed using Prism 6 software. Statistical significance was defined as p < 0.05. (*p < 0.05, **p < 0.01, ***p < 0.001, ^#^p < 0.0001).

## Additional Information

**How to cite this article**: Asano, Y. *et al.* IER5 generates a novel hypo-phosphorylated active form of HSF1 and contributes to tumorigenesis. *Sci. Rep.* **6**, 19174; doi: 10.1038/srep19174 (2016).

## Supplementary Material

Supplementary Information
