# Supplementary material for: IER5 generates a novel hypo-phosphorylated active form of HSF1 and contributes to tumorigenesis
Source: Sci Rep. 2016 Jan 12;6:19174. doi: 10.1038/srep19174 (PMC4709660; doi:10.1038/srep19174)
Supplement: Supplementary Information [file srep19174-s1.pdf]

Supplementary information

IER5 generates a novel hypo-phosphorylated active form of HSF1 and contributes to tumorigenesis

Yoshinori Asano[1,2]#, Tatsuya Kawase[3,4]#, Atsushi Okabe[5], Shuichi Tsutsumi[5], Hitoshi Ichikawa[6], Satoko Tatebe[4], Issay Kitabayashi[7], Fumio Tashiro[4], Hideo Namiki[2], Tadashi Kondo[1], Kentaro Semba[2], Hiroyuki Aburatani[5], Yoichi Taya[3], Hitoshi Nakagama[8] and Rieko Ohki[1]*

[1]Division of Rare Cancer Research, [3]Radiobiology Division, [6]Department of Clinical Genomics, [7]Division of Hematological Malignancy, [8]Division of Cancer Development System, National Cancer Center Research Institute, Tsukiji 5-1-1, Chuo-ku, Tokyo 104-0045, Japan

[2]Graduate School of Advanced Science and Engineering, Waseda University, 3-4-1 Okubo, Shinjuku-ku, Tokyo, 169-8555, Japan

[4]Department of Biological Science and Technology, Faculty of Industrial Science and Technology, Tokyo University of Science, Niijuku 6-3-1, Katsushika-ku, Tokyo 125-8585, Japan

[5]Genome Science Division, Research Center for Advanced Science and Technology, The University of Tokyo, 4-6-1 Komaba, Meguro-ku, Tokyo 153-8904, Japan

Running title: IER5, a novel activator of HSF1 in cancers

Key words: p53, HSF1, HSP family, IER5, super-enhancer

*Correspondence: rohki@ncc.go.jp

# These authors equally contributed to this work.

Supplementary Figure Legends

Fig. S1 *IER5* mRNA is induced by growth stimuli and the *IER5* gene is associated with super-enhancers in cancer cell lines

(A) *IER5* induction by the indicated stimuli in various cell lines were analyzed using the Gene Logic SCIANTIS database (http://www.genelogic.com), a comprehensive genome-wide gene expression database annotated with detailed clinical and biological information. Conditions that resulted in more than a 2-fold increase in microarray expression signal intensities are listed.

(B) Analysis using the COSMIC database (http://cancer.sanger.ac.uk/cancergenome/projects/cosmic/), overexpression of *IER5* mRNA in human cancers was analyzed. Overexpression was defined as samples with a Z-score >2.

(C) The *IER5* gene is associated with super-enhancers in various cancer cell lines. Hnisz et al.[17] reported super-enhancers in the indicated cancer cell lines.

Fig. S2 *HSP* family genes are induced by IER5

(A) Representative images of IER5 mutants used in this study are shown.

(B) H1299 cells were transfected with control, IER5-Flag or mut 1-Flag expression vectors. Cells were harvested 27 hrs post-transfection, and expression of wild type and mutant IER5 proteins were analyzed by Western blotting.

(C, D) H1299 cells were transfected with control, IER5-Flag or mutant IER5-Flag expression vectors. Cells were harvested 27 hrs post-transfection, and *HSPA1A*, *HSPA6* and *DNAJB1* mRNA levels were analyzed by Northern blotting (C), and protein levels of wild type and mutant IER5s were analyzed by Western blotting (D). The expression level of N-terminally deleted mut3 was extremely low, and we therefore did not use this mutant for the downstream experiments.

Fig. S3 IER5 reduces methylation and acetylation of HSF1

Significant reductions in methylation and acetylation levels at E113 and K118 were detected. Frequency of phosphorylation detected by LC-MS/MS analysis is shown.

Fig. S4 Expression of IER5 and HSF1 is required for anchorage-independent cell growth

HeLa cells (A; 2 x 10^3 cells or B; 1 x 10^3 cells) were treated as in Figs. 7A and 7B and cell growth was analyzed on the indicated days. (**p< 0.01, ***p< 0.001, #p<0.0001)

Fig. S5 Expression of IER5 and HSF1 target genes are correlated in cancers, and higher expression of IER5 mRNA is related to poorer prognosis of breast and brain cancer patients

(A) Correlations between mRNA expression levels of *IER5* and HSF1 target genes in bladder cancer (dataset GSE13507) were analyzed.

(B) Relapse-free survival of patients with breast cancer (dataset GSE2990) was analyzed using the PrognoScan database. Correlations between mRNA expression of *IER5* and HSF1 target genes were also analyzed.

(C) Overall survival of patients with brain cancer (astrocytoma, dataset GSE4271-GPL96) was analyzed using the PrognoScan database. Correlation of *IER5* and *HSPA6* expression was also analyzed.

Asano_Fig. S1

A

Cell line	Derivation	Treatment
Daudi	Burkit lymphoma	PMA + Ionomycine, 2h
Jurcat	T-cell leukemia	PMA + Ionomycine, 8h
U937	histiocytic lymphoma	PMA + Ionomycine, 24h
CD4+ T-lymphocyte		anti-CD3 + PMA, 8h
		anti-CD3 + anti-CD28, 8h
		PMA + Ionomycine, 2h
B-lymphocyte		lipopolysaccharide, 2h
		PMA + Ionomycine, 2h
		anti-IgG, 2h
T-lymphocyte		PMA + Ionomycine, 2h
Peripheral blood leukocytes		TNF-alpha + fMLP, 4h
		lipopolysaccharide, 4h
		concanavalin A, 4h

B

	overexpression sample	Total sample	%
Ovary	36	266	13.5
Liver	15	147	10.2
Lung	67	865	7.8
Stomach	20	285	7.0
Endometrium	33	476	6.9
Breast	60	989	6.1
Thyroid	26	494	5.3
Central nervous system	24	480	5.0
Kidney	25	503	5.0
Large intestine	27	579	4.7
Urinary tract	9	198	4.6
Upper aerodigestive tract	18	397	4.5
Pancreas	3	70	4.3
Prostate	7	198	3.5
Haematopoietic and lymphoid	5	173	2.9
Skin	4	336	1.2
Cervix	1	125	0.8

C

Cell line	Tissue Type	Cancer ID	Super enhancer	p53 status
DND41	Blood cancer	T cell leukemia #1		mutant
Jurkat	Blood cancer	T cell leukemia #2		mutant
RPMI-8402	Blood cancer	T cell leukemia #3		mutant
K562	Blood cancer	CML		null
GM12878	Blood cancer	Lymphoblastoid		NA
MM1S	Blood cancer	Multiple myeloma		wild type
u87	Brain cancer	Glioblastoma	yes	wild type
HCC1954	Breast cancer	Breast cancer #1	yes	mutant
MCF-7	Breast cancer	Breast cancer #2	yes	wild type
HeLa	Cervical cancer	Cervical cancer	yes	inactivated
HCT-116	Colrectal cancer	Colorectal cancer #1		wild type
VACO_9m	Colrectal cancer	Colorectal cancer #2		NA
VACO_400	Colrectal cancer	Colorectal cancer #3		mutant
VACO_503	Colrectal cancer	Colorectal cancer #4	yes	NA
HepG2	Liver cancer	Liver cancer		wild type
H2171	Lung cancer	Lung cancer		mutant
Panc1	Pancreatic cancer	Pancreatic cancer		mutant
LnCAP	Prostate cancer	Prostate cancer		wild type

Asano_Fig. S2

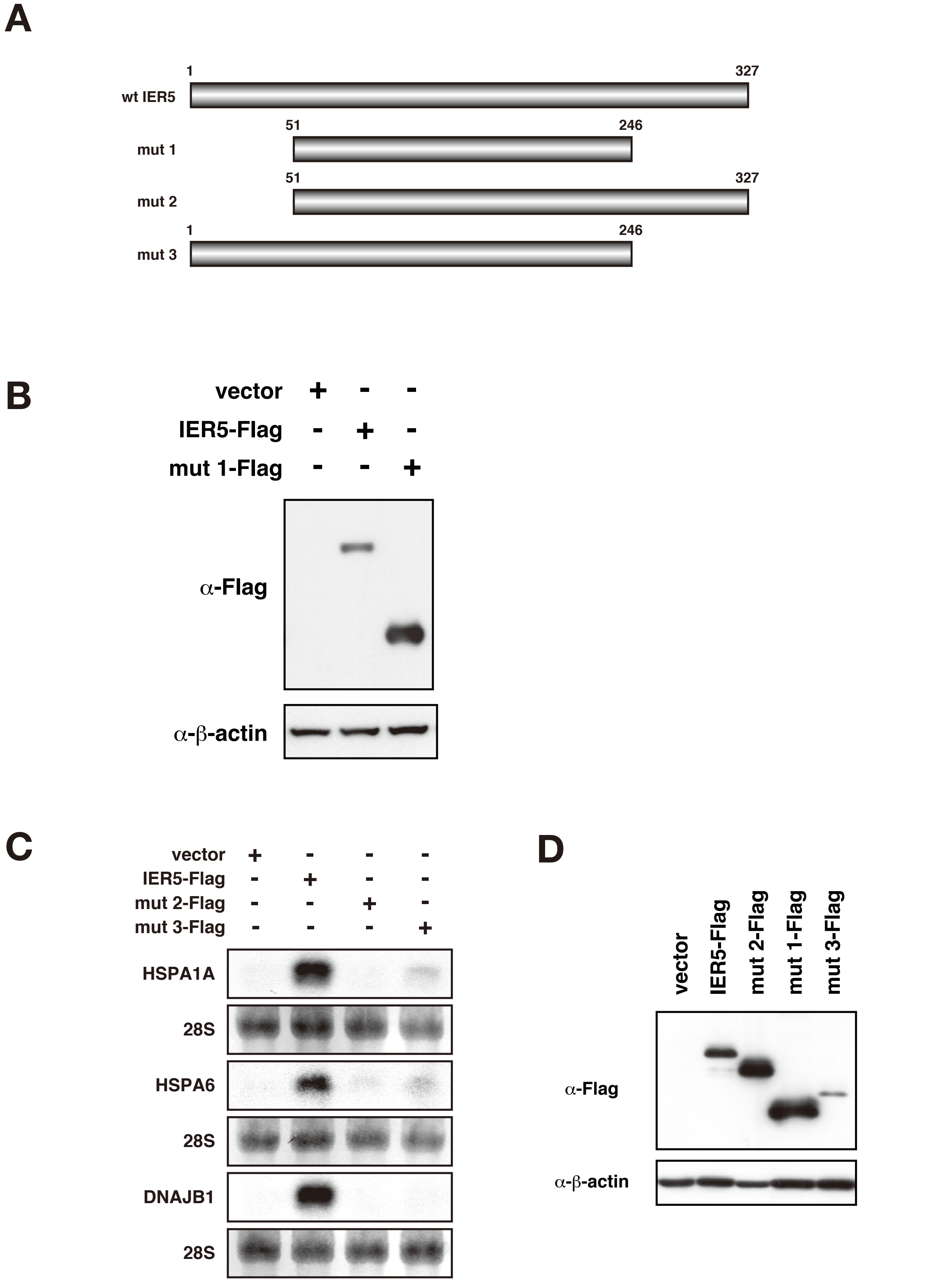
A
1
327
wt IER5
51
246
mut 1
51
327
mut 2
1
246
mut 3
B
vector + - -
IER5-Flag - + -
mut 1-Flag - - +
α-Flag
α-β-actin
C
vector + - - -
IER5-Flag - + - -
mut 2-Flag - - + -
mut 3-Flag - - - +
HSPA1A
28S
HSPA6
28S
DNAJB1
28S
D
vector
IER5-Flag
mut 2-Flag
mut 1-Flag
mut 3-Flag
α-Flag
α-β-actin

Asano_Fig. S3

Sites of decreased methylation or acetylation by IER5

	E113	K118
vector	++	+
IER5	+	-

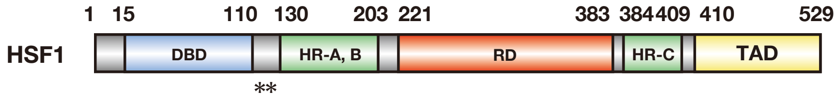

detection of modification

7-9/9	++++
5-6/9	+++
3-4/9	++
1-2/9	+
0/9	-

Asano_Fig. S4

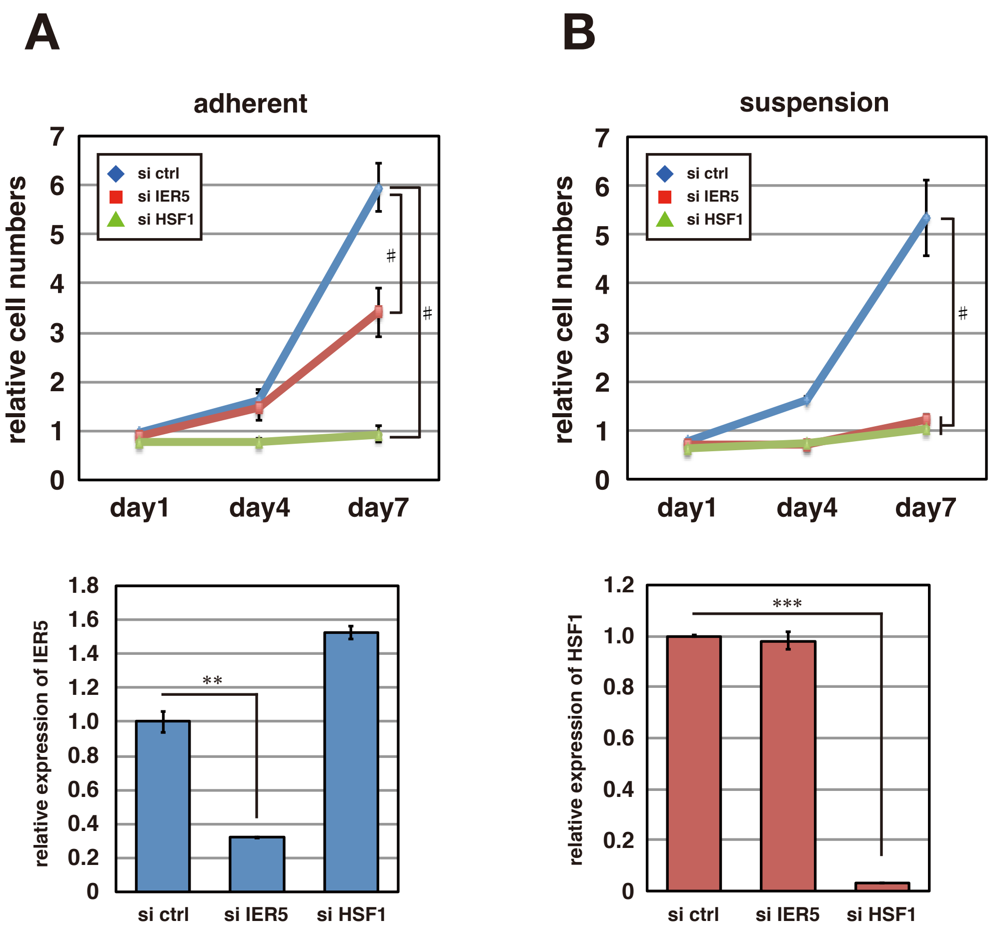

A
adherent
relative cell numbers
si ctrl
si IER5
si HSF1
#
#
day1
day4
day7
relative expression of IER5
**
si ctrl
si IER5
si HSF1
B
suspension
relative cell numbers
si ctrl
si IER5
si HSF1
#
day1
day4
day7
relative expression of HSF1
***
si ctrl
si IER5
si HSF1

Asano_Fig. S5

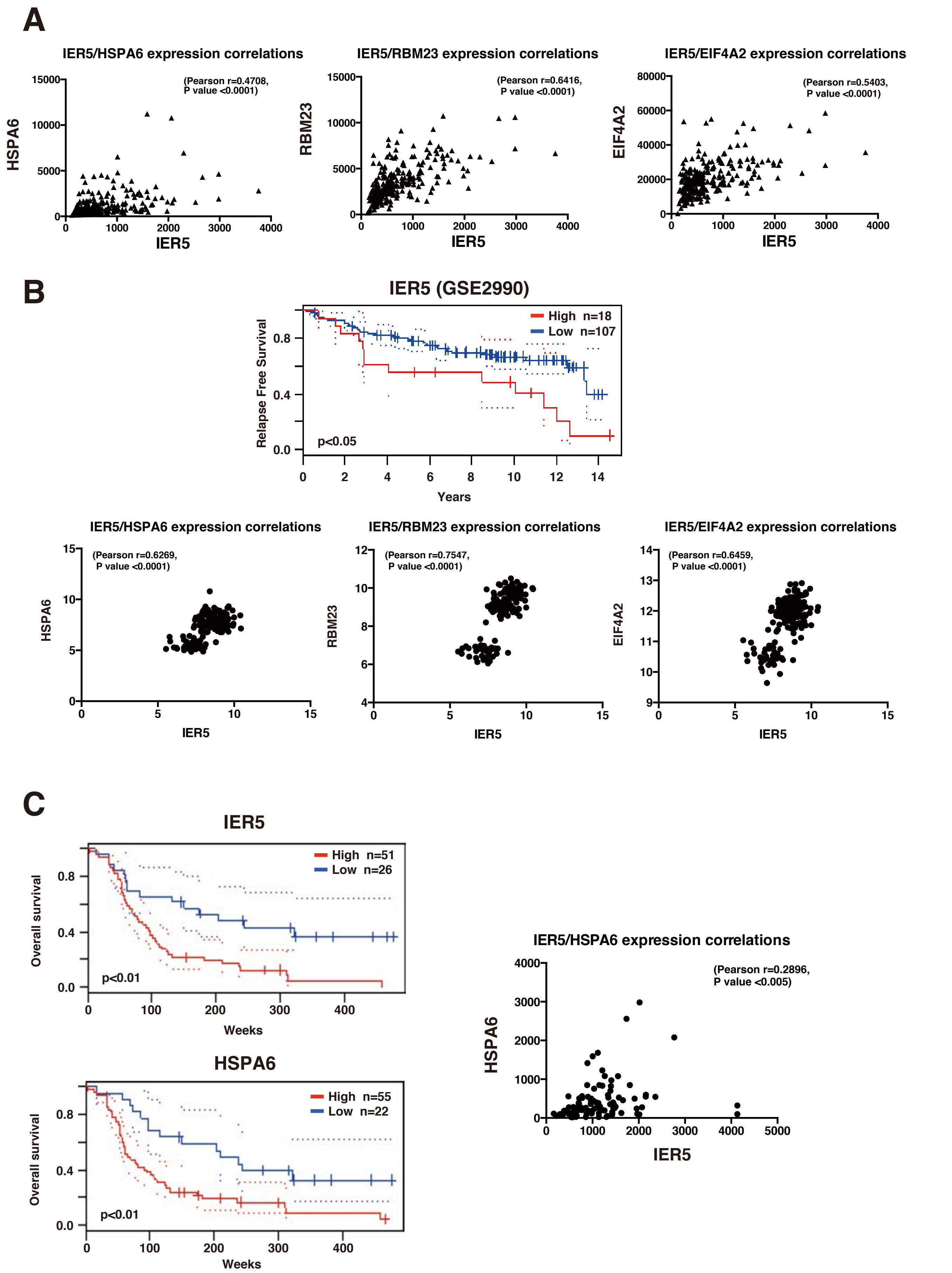
A
IER5/HSPA6 expression correlations
(Pearson r=0.4708, P value <0.0001)
HSPA6
IER5
IER5/RBM23 expression correlations
(Pearson r=0.6416, P value <0.0001)
RBM23
IER5
IER5/EIF4A2 expression correlations
(Pearson r=0.5403, P value <0.0001)
EIF4A2
IER5
B
IER5 (GSE2990)
High n=18
Low n=107
Relapse Free Survival
p<0.05
Years
IER5/HSPA6 expression correlations
(Pearson r=0.6269, P value <0.0001)
HSPA6
IER5
IER5/RBM23 expression correlations
(Pearson r=0.7547, P value <0.0001)
RBM23
IER5
IER5/EIF4A2 expression correlations
(Pearson r=0.6459, P value <0.0001)
EIF4A2
IER5
C
IER5
High n=51
Low n=26
Overall survival
p<0.01
Weeks
HSPA6
High n=55
Low n=22
Overall survival
p<0.01
Weeks
IER5/HSPA6 expression correlations
(Pearson r=0.2896, P value <0.005)
HSPA6
IER5
